# The photodecarboxylative addition of carboxylates to phthalimides as a key-step in the synthesis of biologically active 3-arylmethylene-2,3-dihydro-1*H*-isoindolin-1-ones

**DOI:** 10.3762/bjoc.13.275

**Published:** 2017-12-20

**Authors:** Ommid Anamimoghadam, Saira Mumtaz, Anke Nietsch, Gaetano Saya, Cherie A Motti, Jun Wang, Peter C Junk, Ashfaq Mahmood Qureshi, Michael Oelgemöller

**Affiliations:** 1 James Cook University, College of Science and Engineering, Townsville, Queensland 4811, Australia; 2 Dublin City University, School of Chemical Sciences, Dublin 9, Ireland; 3 Australian Institute of Marine Science, Townsville, Queensland, Australia; 4 Bahauddin Zakariya University, Institute of Chemical Sciences, Multan, Pakistan

**Keywords:** anesthetics, arylmethylenedihydroisoindolinones, photochemistry, photodecarboxylation, phthalimide

## Abstract

The synthesis of various 3-arylmethylene-2,3-dihydro-1*H*-isoindolin-1-ones was realized following a simple three-step process. The protocol utilized the photodecarboxylative addition of readily available carboxylates to *N*-(bromoalkyl)phthalimides as a versatile and efficient key step. The initially obtained hydroxyphthalimidines were readily converted to the desired *N*-diaminoalkylated 3-arylmethylene-2,3-dihydro-1*H*-isoindolin-1-ones via acid-catalyzed dehydration and subsequent nucleophilic substitution with the corresponding secondary amines. The procedure was successfully applied to the synthesis of known local anesthetics (AL-12, AL-12B and AL-5) in their neutral forms.

## Introduction

**Figure 1:**
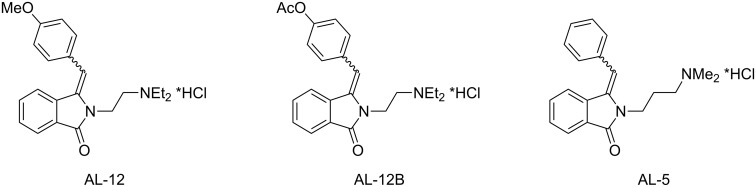
Molecular structures of AL-12, AL-12B and AL-5.

Phthalimides and their related 3-alkyl- and 3-arylmethylene-2,3-dihydro-1*H*-isoindolin-1-ones play an important role in medicinal chemistry due to their biological activities for a wide range of therapeutic applications [1–7]. AL-12, AL-12B and AL-5 (Figure 1), for example, were described as highly active local anesthetics distinctly exceeding the efficiencies of common local anesthetics such as procaine, xylocaine/lidocaine and tetracaine [8]. Their molecular structures contain the three key elements of all local anesthetics: (a) a lipophilic aromatic ring, (b) an amide (or ester) linker, and (c) a terminal tertiary amine [9]. The original synthesis of these bioactive compounds involved a Perkin condensation followed by an amination reaction. An alternative pathway to AL-12B has been described by Couture and co-workers and incorporated an intramolecular Horner–Wadsworth–Emmons reaction as a crucial step [10].

Due to their diverse biological activities, a variety of synthetic pathways to 3-arylmethylene-2,3-dihydro-1*H*-isoindolin-1-ones has been developed over the past two decades [10–22]. The photodecarboxylative addition of phenylacetates to phthalimides represents a mild alternative entrance to these target molecules [23–31]. The reaction utilizes phenylacetate salts as readily available alkylation agents [32–33]. Selected transformations have been furthermore realized on large multigram scales [25,34–35] and in continuous-flow mode [36–40]. The photodecarboxylation procedure was subsequently applied to the synthesis of 2-dialkylaminoalkyl-3-arylmethylene-2,3-dihydro-1*H*-isoindolin-1-ones.

**Scheme 1:**
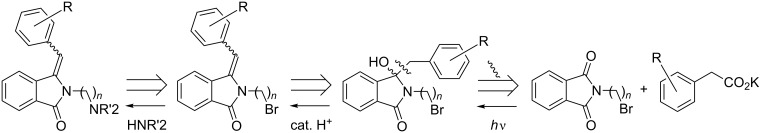
Retrosynthetic analysis of AL-12, AL-12B and AL-5 (in their neutral forms) and their derivatives.

The retrosynthetic analysis is depicted in Scheme 1. The initial step comprises the photodecarboxylative addition of phenylacetate to commercially available *N*-(bromoalkyl)phthalimides, yielding the corresponding benzylated hydroxyphthalimidine derivatives as key intermediates. Subsequent acid-catalyzed dehydration [41], followed by amination [42] furnishes the desired target compounds. The amino group is introduced in the final step as it would otherwise interfere with the desired photoreaction. In fact, amines are very potent electron donors and are easily oxidized by the excited phthalimide chromophore [43–46].

## Results and Discussion

**Scheme 2:**
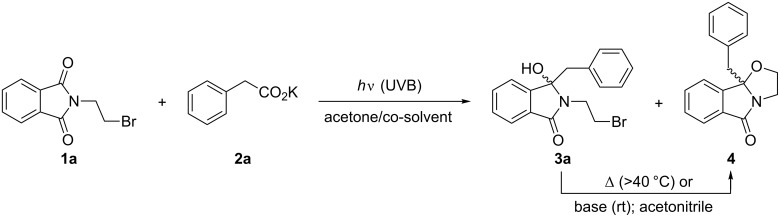
Optimization study using phenylacetate **2a**.

Initial irradiation experiments were conducted with *N*-(2-bromoethyl)phthalimide (**1a**) and potassium phenylacetate (**2a**) as a model system (Scheme 2 and Table 1). The salt **2a**, generated from the corresponding phenylacetic acid and potassium carbonate, was used in excess amounts to suppress competing ‘simple’ decarboxylation (-CO_2_^−^ ↔ -H exchange) reactions to the corresponding toluene derivatives [25].

**Table 1:** Impact of the reaction medium on the photodecarboxylative addition.

Entry	Co-solvent	*T* [°C]	Time [h]	Yield [%]

1^a^	water	30	4	45^b^ (**3a**)
2^a^	water	20	4	49^b^ (**3a**)
3	pH 7 buffer	20	3	87 (**3a**)

^a^Compound **4** formed as byproduct (25–30%). ^b^Isolated yield after column chromatography.

Following the established protocol and utilizing a 1:1 acetone/water mixture as reaction medium [27,30], irradiations with UVB light (300 ± 30 nm) for 4 hours under nitrogen purging in Pyrex flasks (cutoff: <300 nm [47]) furnished the desired addition product **3a** in yields of 45 and 49% (Table 1, entries 1 and 2). The pH raised from approx. 7.5 at the beginning to about 11.5 at the end of each irradiation experiment. In both cases, the tetracyclic oxazolidine compound **4**, originating from intramolecular nucleophilic substitution, was obtained as a byproduct in 25–30%. Noteworthy, compound **4** is formed as the only product when **1a** is treated with organometallic reagents [48–50]. When the photoreaction was repeated in a 1:1 mixture of acetone and pH 7 buffer at 20 °C [28], the formation of **4** was prevented and the desired benzylated hydroxyphthalimidine **3a** precipitated during irradiation for 3 hours. Subsequently, **3a** was obtained in an excellent yield of 87% by simple filtration and washing (Table 1, entry 3). The pH increased from about 7.8 at the start to ca. 8.7 at the end of the irradiation. Temperature control was crucial as thermal conversion of **3a** into **4** was found to occur above 40 °C. Compound **1a** could be converted quantitatively to the oxazolidine derivative **4** by treatment with either sodium carbonate or potassium *tert*-butoxide in acetonitrile.

**Scheme 3:**
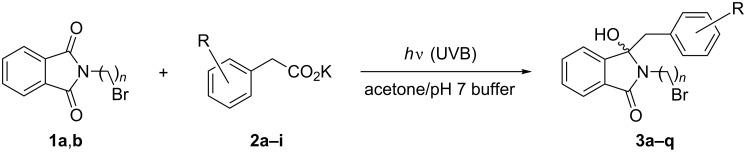
Photodecarboxylative additions to *N*-(bromoalkyl)phthalimides.

Mixtures of *N*-(bromoalkyl)phthalimides **1** and phenylacetates **2** in acetone/pH 7 buffer were subsequently irradiated with UVB light for 2–4 hours (Scheme 3 and Table 2). With the *N*-(2-bromoethyl)- and *N*-(3-bromopropyl)phthalimides **1a** and **1b**, the desired benzylated products **3a–q** were obtained as colorless crystalline solids in good to excellent yields of 63–95% (Table 2, entries 1–17). In many cases, the photoproducts **3** simply precipitated during irradiation or after removal of the co-solvent acetone and could be isolated by filtration. In all other cases, the desired products **3** were obtained after extraction and subsequent column chromatography. All compounds **3** showed a characteristic pair of doublets between 3 and 4 ppm with a large geminal *^2^J* coupling of 12–16 Hz for the benzylic methylene group (-C*H*_2_Ar) in their ^1^H NMR spectra and a singlet at 90 ± 3 ppm for the newly formed tertiary alcohol (*C*–OH) in their ^13^C NMR spectra, respectively. Small amounts (<10%) of non-volatile toluene derivatives were occasionally detected in the crude products by ^1^H NMR spectroscopic analysis but no attempts were made to isolate these compounds. The reaction was additionally studied with natural sunlight [51–52]. Solutions of **1a** and **2a** were exposed to direct sunlight in a solar float developed by Liu and co-workers [53–54]. After 6 hours of illumination, the reaction had reached a conversion of 47% and **3a** was subsequently isolated by column chromatography in 26% yield (Table 2, entry 18).

**Table 2:** Experimental results for photodecarboxylative additions.

Entry	*n*	R	Time [h]	Yield **3** [%]

1	2 (**1a**)	H (**2a**)	3	87 (**3a**)
2	2 (**1a**)	4-F (**2b**)	3	83 (**3b**)
3	2 (**1a**)	4-Cl (**2c**)	3	79 (**3c**)
4	2 (**1a**)	4-Br (**2d**)	3	63 (**3d**)
5	2 (**1a**)	4-MeO (**2e**)	3	85 (**3e**)
6	2 (**1a**)	4-Me (**2f**)	3	88 (**3f**)
7	2 (**1a**)	3-Me (**2g**)	3	66 (**3g**)
8	2 (**1a**)	2-Me (**2h**)	3	71 (**3h**)
9	2 (**1a**)	4-AcO (**2i**)	2	77 (**3i**)
10	3 (**1b**)	H (**2a**)	3	95 (**3j**)
11	3 (**1b**)	4-F (**2b**)	3	75 (**3k**)
12	3 (**1b**)	4-Cl (**2c**)	3	80 (**3l**)
13	3 (**1b**)	4-Br (**2d**)	3	73 (**3m**)
14	3 (**1b**)	4-MeO (**2e**)	3	76 (**3n**)
15	3 (**1b**)	4-Me (**2f**)	3	89 (**3o**)
16	3 (**1b**)	3-Me (**2g**)	3	91 (**3p**)
17	3 (**1b**)	2-Me (**2h**)	3	73 (**3q**)
18^a^	2 (**1a**)	H (**2a**)	6	26 (**3a**)^b^
19	1 (**1c**)	H (**2a**)	4	37 (**5**)

^a^Exposure to sunlight in a solar float. ^b^47% conversion of **1a**.

**Figure 2:**
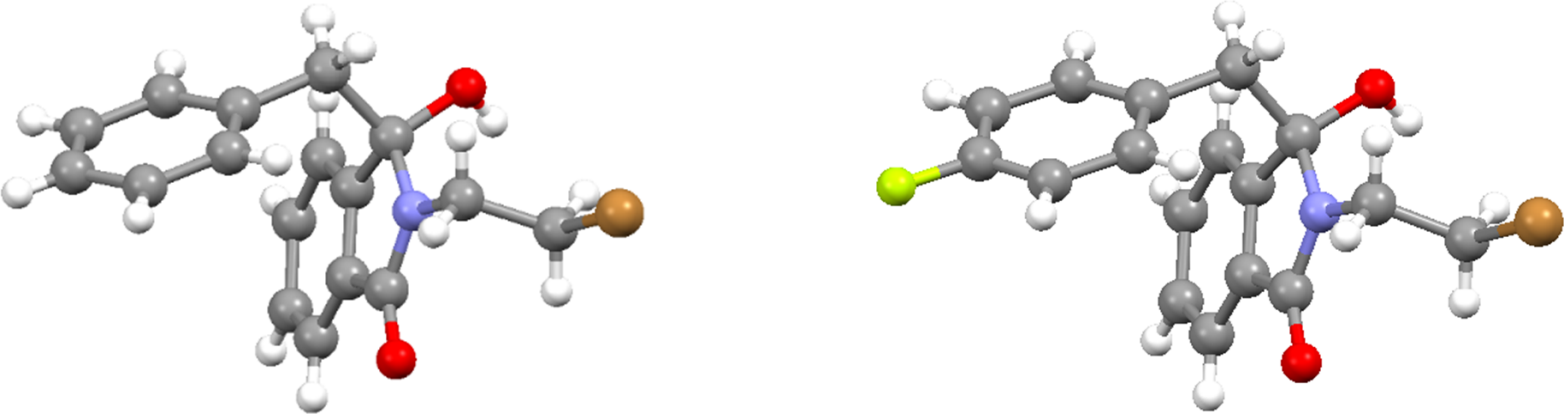
Crystal structures of photoaddition products **3a** (left) and **3b** (right).

The structures of the photoaddition products **3a** and **3b** were unambiguously confirmed by X-ray crystallography (Figure 2). In the solid state, molecules of both compounds undergo hydrogen bonding between the newly formed hydroxy group and the intact carbonyl group, resulting in a one-dimensional network (see Supporting Information File 1).

**Scheme 4:**
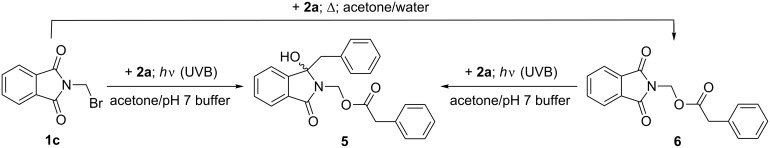
Formation of **5** by photodecarboxylative additions.

Notably, irradiation of *N*-(bromomethyl)phthalimide (**1c**) in the presence of phenylacetate (**2a**) did not furnish the desired addition product, but the benzylated ester **5** in 37% yield instead (Scheme 4). The electron-withdrawing character of the phthalimide group favored thermal nucleophilic substitution between **1c** and phenylacetate (**2a**) to phthalimide **6**. Ester **6** was indeed obtained in 37% yield by gently heating a mixture of phthalimide **1c** and phenylacetate (**2a**) in acetone/water. Subsequently, compound **5** was independently prepared by photodecarboxylative benzylation of **6** for 4 h in 91% yield.

**Scheme 5:**
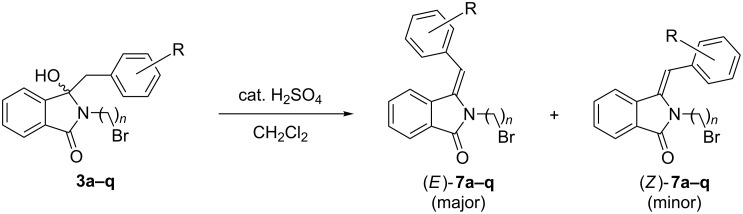
Acid-catalyzed dehydration of **3a–q**.

Sulfuric acid-catalyzed dehydrations of the benzylated hydroxyphthalimidines **3a–q** in dichloromethane at room temperature resulted in the corresponding olefins **7a–q** in good to excellent yields of 66–95% (Scheme 5 and Table 3). The simple reaction protocol enabled parallel operations in a Radleys Carousel 6 Plus Reaction Station™. In line with DFT calculations by Kise et al. [12] and independently by Li and Janesko [21], the thermodynamically favored *E*-isomer was obtained as the main or sole product. The high *E*-selectivity was furthermore confirmed by ^1^H NMR analyses. The olefinic protons of the minor *Z*-isomers are shifted downfield by approx. 0.25 ppm due to the shielding effect of the adjacent isoindolin-1-one ring [31,41]. A similar but deshielding effect was found for the *N*-bromoalkyl protons and the arylmethylene group. No such shifts were observed for the corresponding major *E*-isomers.

**Table 3:** Experimental results for acid-catalyzed dehydrations.

Entry	*n*	R	*E*/*Z* ratio^a^	Yield **7** [%]

1	2	H	9:1	91 (**7a**)
2	2	4-F	>10:1	85 (**7b**)
3	2	4-Cl	>10:1	88 (**7c**)
4	2	4-Br	>10:1	83 (**7d**)
5	2	4-MeO	>10:1	90 (**7e**)
6	2	4-Me	>10:1	94 (**7f**)
7	2	3-Me	>10:1	83 (**7g**)
8	2	2-Me	>10:1	90 (**7h**)
9	2	4-AcO	8:1	66 (**7i**)
10	3	H	>10:1	83 (**7j**)
11	3	4-F	>10:1	91 (**7k**)
12	3	4-Cl	>10:1	90 (**7l**)
13	3	4-Br	>10:1	84 (**7m**)
14	3	4-MeO	>10:1	95 (**7n**)
15	3	4-Me	>10:1	92 (**7o**)
16	3	3-Me	>10:1	92 (**7p**)
17	3	2-Me	>10:1	83 (**7q**)

^a^Determined by ^1^H NMR analysis.

**Figure 3:**
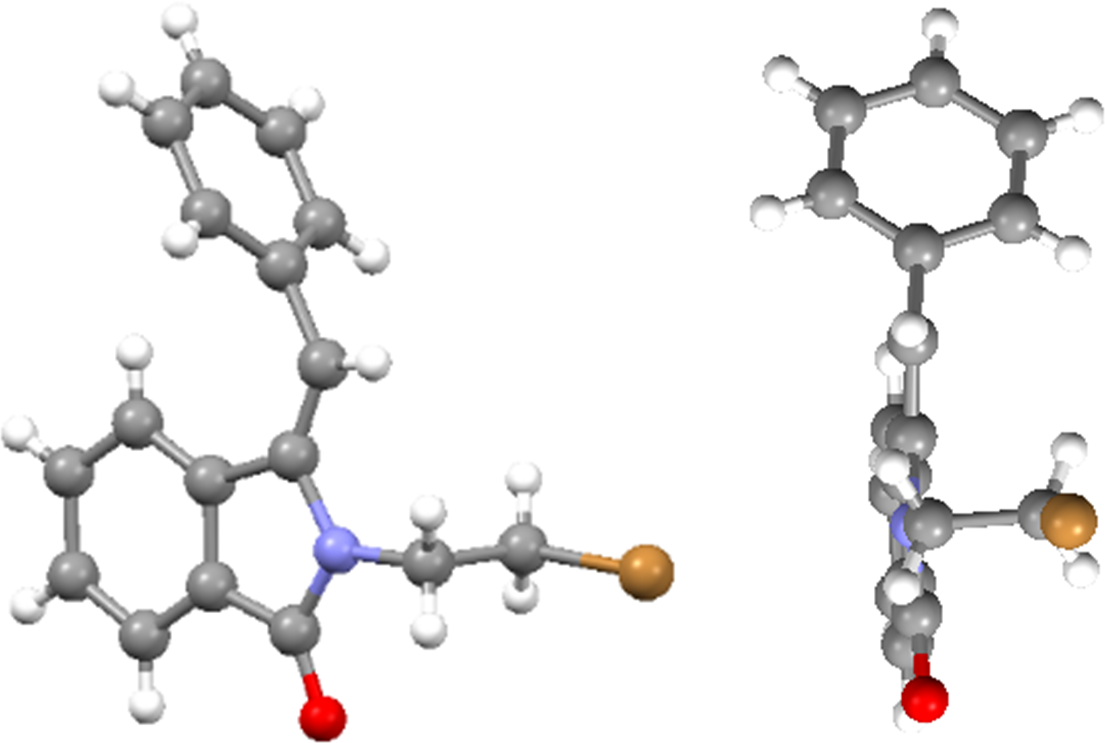
Crystal structure of (*E*)**-7a**. Side view and front view.

The structure of the dehydration product (*E*)-**7a** was furthermore confirmed by X-ray crystallographic analysis (Figure 3). Remarkably, the phenyl group of the arylmethylene unit is positioned almost perpendicular to the isoindolinone ring. Compound (*E*)-**7a** forms dimers through CH–π interactions between the phenyl ring and the olefinic =CH group (see Supporting Information File 1).

**Scheme 6:**
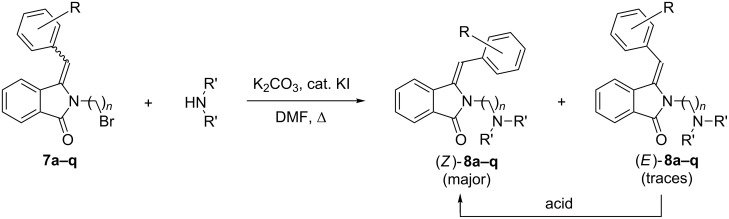
Amination of dehydrated products **7a–q**.

The final amination step was achieved following a modified procedure by Marzabadi and co-workers [42]. Catalytic amounts of potassium iodide were added for in situ halogen exchange. The dehydrated products **7a–q** were heated in the presence of the respective secondary amine, K_2_CO_3_ and KI in DMF (Scheme 6 and Table 4). Subsequent work-up and isolation by column chromatography furnished the desired 2-dialkylaminoalkyl-3-arylmethylene-2,3-dihydro-1*H*-isoindolin-1-ones **8a–y** in moderate yields of 49–61% (Table 4), among these the biologically active AL-12 (**8e**), AL-12A (**8i**) and AL-5 (**8w**) in their neutral forms. Interestingly, the *Z*-isomer of **8a–y** was formed as the sole product in almost all cases as confirmed by ^1^H NMR analyses and by comparison with literature data (Table 4, entries 1–25) [10]. Subsequent investigations revealed that *E*-to-*Z* isomerization was caused during acidic work-up or purification. When products **8a** and **8j** were isolated by extraction under neutral conditions, the high *E*-selectivity of the dehydration step was retained. After column chromatographic purification on silica gel, partial isomerization to the *Z*-isomers was again observed (Table 4, entries 26 and 27).

**Table 4:** Experimental results for amination reactions.

Entry	*n*	R	R’	*E*/*Z* ratio^a^	Yield **8** [%]

1 (**7a**)	2	H	C_2_H_5_	>1:10	55 (**8a**)
2 (**7b**)	2	4-F	C_2_H_5_	>1:10	55 (**8b**)
3 (**7c**)	2	4-Cl	C_2_H_5_	>1:10	53 (**8c**)
4 (**7d**)	2	4-Br	C_2_H_5_	>1:10	51 (**8d**)
5 (**7e**)	2	4-MeO	C_2_H_5_	>1:10	50 (**8e**)
6 (**7f**)	2	4-Me	C_2_H_5_	>1:10	57 (**8f**)
7 (**7g**)	2	3-Me	C_2_H_5_	>1:10	51 (**8g**)
8 (**7h**)	2	2-Me	C_2_H_5_	>1:10	58 (**8h**)
9 (**7i**)	2	4-AcO	C_2_H_5_	>1:10	57 (**8i**)
10 (**7j**)	3	H	C_2_H_5_	>1:10	53 (**8j**)
11 (**7k**)	3	4-F	C_2_H_5_	>1:10	57 (**8k**)
12 (**7l**)	3	4-Cl	C_2_H_5_	>1:10	60 (**8l**)
13 (**7m**)	3	4-Br	C_2_H_5_	>1:10	59 (**8m**)
14 (**7n**)	3	4-MeO	C_2_H_5_	>1:10	55 (**8n**)
15 (**7o**)	3	4-Me	C_2_H_5_	>1:10	58 (**8o**)
16 (**7p**)	3	3-Me	C_2_H_5_	>1:10	61 (**8p**)
17 (**7q**)	3	2-Me	C_2_H_5_	>1:10	49 (**8q**)
18 (**7a**)	2	H	CH_3_	>1:10	58 (**8r**)
19 (**7b**)	2	4-F	CH_3_	>1:10	57 (**8s**)
20 (**7c**)	2	4-Cl	CH_3_	>1:10	55 (**8t**)
21 (**7e**)	2	4-MeO	CH_3_	>1:10	49 (**8u**)
22 (**7f**)	2	4-Me	CH_3_	>1:10	60 (**8v**)
23 (**7j**)	3	H	CH_3_	>1:10	58 (**8w**)
24 (**7k**)	3	4-F	CH_3_	>1:10	55 (**8x**)
25 (**7q**)	3	2-Me	CH_3_	>1:10	53 (**8y**)
26 (**7a**)	2	H	C_2_H_5_	>10:1^b^/1:1^c^	65 (**8a**)
27 (**7j**)	3	H	C_2_H_5_	>10:1^b^/10:7^c^	72 (**8j**)

^a^After acidic work-up. Determined by ^1^H NMR analysis. ^b^Crude product after neutral work-up. ^c^Pure product after neutral work-up followed by column chromatography on silica gel.

**Figure 4:**
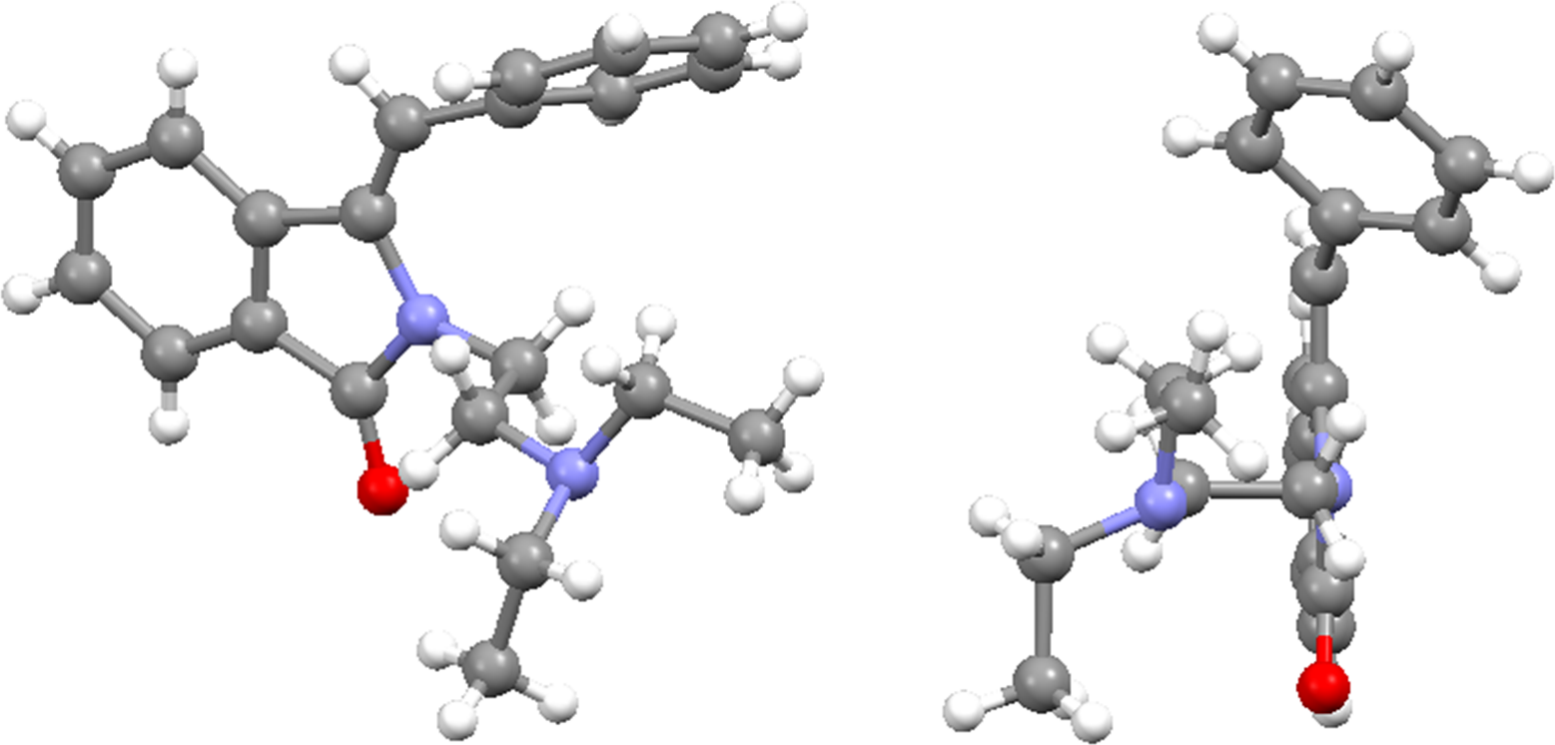
Crystal structure of (*Z*)-**8a**. Side view and front view.

The solid state structure of the amination product (*Z*)-**8a** was furthermore established by X-ray crystal structure analysis (Figure 4). The phenyl group of the arylmethylene unit is positioned almost perpendicular to the isoindolinone ring on top of one of the *N*-ethyl groups of the side chain.

**Scheme 7:**
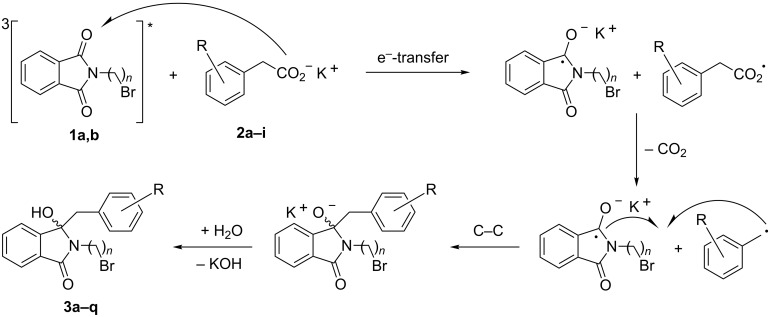
Mechanistic scenario for the photodecarboxylative addition.

The mechanism of the photodecarboxylation is well established (Scheme 7) and involves triplet sensitization by acetone and electron transfer between the phenylacetate and the excited phthalimide [55–57]. Subsequent decarboxylation, radical combination and protonation furnishes the observed benzylated hydroxyphthalimidine derivatives **3a–q**.

**Scheme 8:**
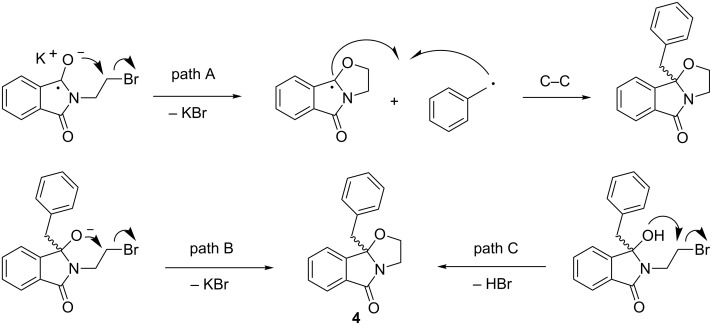
Possible scenarios for nucleophilic cyclization to **4**.

The nucleophilic cyclization to the oxazolidine derivative **4** may occur at different stages of the photodecarboxylation (Scheme 8). The phthalimide radical anion may undergo nucleophilic cyclization prior to C–C bond formation (path A), as postulated by Griesbeck et al. for the photocyclization of phthaloyl-L-methionine [58]. Alternatively, the alcoholate obtained after C–C bond formation may cyclize to the oxazolidine (path B), as known from reactions of **1a** with organometallic reagents [48–50]. When compound **1a** was treated with either sodium carbonate or potassium *tert*-butoxide it was indeed converted quantitatively to the oxazolidine derivative **4**. Similarly, the final benzylated hydroxyphthalimidine may undergo cyclization instead (path C), as was found to occur at elevated temperature of >40 °C. Temperature control during the reaction, work-up and isolation was thus important to suppress this undesired transformation. The usage of pH 7 buffer as a co-solvent significantly improved yields and selectivity, possibly due to the avoidance of extreme basic conditions found for photodecarboxylations in acetone/water [34,59].

**Scheme 9:**
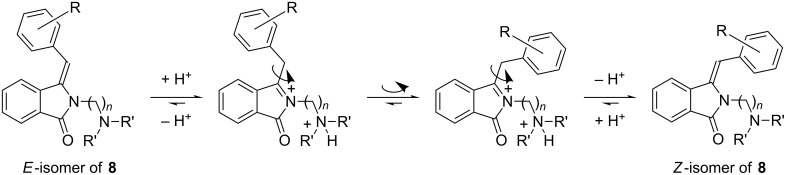
Possible *E*/*Z* isomerization for compounds **8a–y**.

The high stereoselectivity during the formation of compounds **7a–q** is supported by the higher stability of the *E*-isomer, as shown for other simple *N*-substituted benzylideneisoindolin-1-ones [12,21]. Remarkably, acidic reaction conditions induced thermal isomerization of compounds **8a–y**, possibly via an acylaminium cation intermediate (Scheme 9) [60]. The change in stability may be caused by a stabilizing ionic–π interaction for the *Z*-isomer during isomerization [61]. A similar thermal isomerization towards the more stable isomer induced by catalytic amounts of pyridinium *p*-toluenesulfonate has been recently reported by Kise and co-workers [12].

## Conclusion

Photodecarboxylation reactions are emerging as versatile transformations in organic synthesis [24,33,62–65]. The photodecarboxylative addition of phenylacetates to *N*-(bromoalkyl)phthalimides was used as a mild key step in the synthesis of 2-dialkylaminoalkyl-3-arylmethylene-2,3-dihydro-1*H*-isoindolin-1-ones, among these the potent local anesthetics AL-12, AL-12A and AL-5 (in their neutral forms). The simple procedures make this three-step process attractive for in-series continuous flow applications [40,66–67].

## Supporting Information

File 1 Experimental details, detailed spectroscopic and crystallographic data.
