# Supplementary material for: The photodecarboxylative addition of carboxylates to phthalimides as a key-step in the synthesis of biologically active 3-arylmethylene-2,3-dihydro-1H-isoindolin-1-ones
Source: Beilstein J Org Chem. 2017 Dec 20;13:2833–41. doi: 10.3762/bjoc.13.275 (PMC5753101; doi:10.3762/bjoc.13.275)
Supplement: File 1 — Experimental details, detailed spectroscopic and crystallographic data. [file Beilstein_J_Org_Chem-13-2833-s001.pdf]

Supporting Information

for

The photodecarboxylative addition of carboxylates to phthalimides as a key-step in the synthesis of biologically active 3-arylmethylene-2,3-dihydro-1*H*-isoindolin-1-ones

Ommid Anamimoghadam[1], Saira Mumtaz[1], Anke Nietsch[2], Gaetano Saya[1], Cherie A. Motti[3], Jun Wang[1], Peter C. Junk[1], Ashfaq Mahmood Qureshi[4] and Michael Oelgemöller*[1]

Address: [1]James Cook University, College of Science and Engineering, Townsville, Queensland 4811, Australia, [2]Dublin City University, School of Chemical Sciences, Dublin 9, Ireland, [3]Australian Institute of Marine Science, Townsville, Queensland, Australia and [4]Bahauddin Zakariya University, Institute of Chemical Sciences, Multan, Pakistan

Email: Michael Oelgemöller - michael.oelgemoeller@jcu.edu.au

*Corresponding author

Experimental details, detailed spectroscopic and crystallographic data

Experimental

General methods

Solvents and reagents

All solvents and reagents were commercially available (Sigma-Aldrich or Alfa Aesar) and were used without purification. Buffer solutions were prepared from pH 7 Fixanal cartridges and were stored in the dark in a fridge.

Photochemical equipment

Irradiation experiments were carried out in a Rayonet RPR-200 photochemical chamber reactor (Southern New England Ultraviolet Company, USA) equipped with 16 × 8 W RPR-3000Å fluorescent tubes. All reactions were performed in Pyrex glassware.

Analytical methods

Melting point: Melting points were measured using a Tathastu melting point apparatus and are uncorrected.

pH: pH values were recorded on a Jenway 3510 pH meter. The device was calibrated before every usage with 3 standard buffer solutions of pH 4, pH 7 and pH 10.

IR: Infrared spectra were recorded on a Perkin Elmer Spectrum One FT-IR Spectrometer as solids or thin films, and were recorded in the range 600–4000 cm^{-1}. IR peaks are listed in wavenumbers ($\tilde{\nu}$; in cm^{-1}).

NMR: NMR spectra were recorded on an Oxford 300 (^{1}H: 300 MHz and ^{13}C: 75 MHz) with a ^{UNITY}INOVA system console using the Varian Software VnmrJ program or a Bruker AV300 (^{1}H: 300 MHz and ^{13}C: 75 MHz) Nuclear Magnetic Resonance spectrometer with dual probe operating TopSpin 2.1 software. Residual solvent

peaks served as an internal standard [1] and samples were prepared in $CDCl_3$ (δ = 7.26/77.3 ppm) or acetone-d_6 (δ = 2.09/30.6 ppm).

FTMS: High-resolution mass spectroscopic data were determined on a Bruker BioApex 47 FT mass spectrometer with an electospray (ESI) Analytica of Branford source. Ions were detected in positive mode and/or negative mode within a mass range of *m*/*z* 50–2000. Direct infusion of sample (0.2 mg mL^{-1}) was carried out using a Cole Palmer 74900 syringe pump at a flow rate of 100 µl h^{-1}. N_2 (sourced from a Domnick Hunter UHPLCMS18 Nitrogen Generator, flow of 3 L/min and maintained at 200 ºC) was used as the drying gas to assist in desolvation of the droplets produced by ESI from an on axis grounded needle directed to a metal capped nickel coated glass capillary, approximately 1 cm away. All experimental event sequences were controlled and data reduction performed using Bruker Daltonics XMASS ver. 7.0.3.0 software. Detection was in the direct mode from time domain data sets of 512 k (16 scans per experiment). Each spectrum was subjected to zerofill, Gaussian multiplication, and fast Fourier transform and displayed in magnitude mode. The instrument was calibrated using a methanolic solution of sodium trifluoroacetate (0.1 mg/mL MeOH), 200–2000 *m*/*z*.

X-ray crystallographic analyses: These were performed on a Bruker APEX-II CCD diffractometer [2]. Crystallographic data (excluding structure factors) for the structures reported in this paper have been deposited with the Cambridge Crystallographic Data Centre as supplementary numbers CCDC1417120 (**3a**), CCDC1417119 (**3b**), CCDC1417121 (***E*-7a**) and CCDC1417118 (***Z*-8a**). Copies of the data can be obtained free of charge on application to CCDC, 12 Union Road, Cambridge, CB2 1EZ, UK (fax: +44 (0)1223 336033; email: deposit@ccdc.cam.ac.uk).

Chromatographic methods

Separations: Column chromatography was carried out in Pyrex glass columns using Scharlan silica gel 60 (particle size 0.06–0.2 nm) 70–230 mesh ASTM.

TLC: Thin layer chromatography was performed in glass jars on Macherey-Nagel polygram sil G/UV_{254}. Mixtures of ethyl acetate and cyclohexane or ethyl acetate and *n*-hexane were used as mobile phase.

Experimental procedures

Synthesis of (1,3-dioxoisoindolin-2-yl)methyl 2-phenylacetate (6)

1.02 g (4.2 mmol) of *N*-(bromomethyl)phthalimide (**1c**), 1.09 g (8.0 mmol) of phenylacetic acid and 0.56 g (4.0 mmol) of potassium carbonate were mixed in 120 mL of a mixture of acetone and distilled water (1:1 vol %). The solution was heated for 5 h to approx. 70 °C. After evaporation of acetone, the product precipitated as a colorless solid. Filtration, washing with water and drying gave 465 mg (1.6 mmol; 37%) of **6** as a colorless solid.

Photodecarboxylative addition

A mixture of 15 mmoles of phenylacetic acid derivative, 7.5 mmoles of K_2CO_3 in 5 mL of acetone and 15 mL of buffer (pH 7) was added to 5 mmoles of *N*-(bromoalkyl)phthalimide in 10 mL of acetone in a Pyrex Schlenk flask, followed by 70 mL of a 1:1 mixture of acetone:pH 7 buffer. The mixture was sonicated for 10 min and irradiated in a Rayonet chamber reactor while a continuous slow stream of N_2 is passed through the reaction mixture. The progress of the reaction was monitored by TLC or by passing the leaving gas stream through a saturated $Ba(OH)_2$ solution. When all phthalimide was consumed or when precipitation of $BaCO_2$ had ceased, the reaction was stopped. In cases where the product had precipitated during irradiation,

the product was isolated by filtration, washed with water and *n*-hexane and dried. In all other case, most of the acetone was removed via rotary evaporation at low temperatures (water bath <35 °C). In cases where the product precipitated on standing it was isolated by filtration and treated as described above. In all other cases, the reaction mixture was extracted with CH_2Cl_2 (3 × 40 mL) and washed with saturated $NaHCO_3$ (2 × 40 mL) and brine (1 × 40 mL). After drying over $MgSO_4$, the reaction mixture was evaporated to dryness on a rotary evaporator at low temperatures (<35 °C). When necessary, the crude products were purified by column chromatography using mixtures of ethyl acetate and cyclohexane or ethyl acetate and *n*-hexane, respectively.

Solar exposure: Mixtures of *N*-(2-bromoethyl)phthalimide (0.2 mmoles), phenylacetic acid (0.6 mmoles) and K_2CO_3 (0.3 mmoles) in 15 mL of a 1:1 mixture of acetone:pH 7 buffer were filled into three Pyrex test tubes and placed in a solar float. The float was placed inside a large container filled with ice water. The holding frame inside the float kept the test tubes half submerged in water. The reaction mixtures were exposed to direct sunlight under sunny conditions for 6 hours. The workup was the same as described above for irradiations in a laboratory chamber reactor.

Dehydrations

Selected reactions were conducted in a Radleys Carousel 6 Plus Reaction Station™. 4 mmoles of the photoproduct **3a–q** were dissolved in 30 mL of CH_2Cl_2. Approximately 3 drops of concentrated H_2SO_4 were added and the solution was stirred at room temperature. After 3–5 hours, 30 mL of water were added and the organic layer was separated, washed with saturated $NaHCO_3$ (2 × 40 mL) and brine (1 × 40 mL) and dried over $MgSO_4$. After gravity filtration, the filtrate was evaporated to dryness by rotary evaporation. When necessary, the crude products were purified

by column chromatography using mixtures of ethyl acetate and cyclohexane or ethyl acetate and *n*-hexane, respectively.

Aminations

Selected reactions were performed in a Radleys Carousel 6 Plus Reaction Station™. A mixture of the respective amine (3.3 mmoles), dehydrated product **7a–p** (3 mmoles), KI (a tip of a spatula) and K_2CO_3 (1.5 mmoles) in 10 mL DMF was heated to 100 °C for 3 hours. After cooling down to room temperature, an excess of water was added and the solution was acidified using 1 M HCl. The solution was subsequently washed with ethyl acetate (50 mL). The aqueous layer containing the target compound was then basified to pH 10 using ammonia solution and extracted with CH_2Cl_2 (3 × 50 mL). The combined organic phase was collected, washed with brine (1 × 50 mL) and dried over $MgSO_4$. The dried organic layer was evaporated to dryness by rotary evaporation. When necessary, the crude products were purified by column chromatography using mixtures of ethyl acetate and cyclohexane, ethyl acetate and *n*-hexane or ethylacetate, *n*-hexane and NEt_3 (50:49:1 vol %), respectively. For workup under neutral conditions, 30 ml of water were added and the reaction mixture was stirred for 30 min. The solution was extracted with CH_2Cl_2 and further treated as described above.

Spectroscopic data

(1,3-Dioxoisoindolin-2-yl)methyl 2-phenylacetate (6)

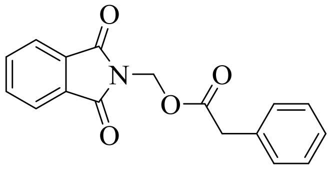

Appearance: colorless solid.

Melting point: 103°C.

IR (film): $\tilde{\nu}$ (cm^{-1}) = 1738, 1166, 977, 726 and 705.

^{1}H-NMR (300 MHz, $CDCl_3$): δ (ppm): 3.64 (s, 2 H, CH_2Ph), 5.75 (s, 2 H, NCH_2), 7.07-7.43 (m, 5 H, CH), 7.79 (m, 2 H, CH), 7.93 (m, 2 H, CH).

^{13}C-NMR (75 MHz, $CDCl_3$): δ (ppm): 41.1 (t, 1 C, CH_2Ph), 61.5 (t, 1 C, NCH_2), 124.3 (s, 1 C, Cq), 127.6 (d, 1 C, CH), 128.9 (d, 2 C, CH), 129.6 (d, 2 C, CH), 132.1 (d, 2 C, CH), 133.5 (s, 2 C, Cq), 135.0 (d, 2 C, CH), 167.0 (s, 2 C, C=O), 170.8 (s, 1 C, C=O).

Photodecarboxylative addition

3-Benzyl-2-(2-bromoethyl)-3-hydroxy-2,3-dihydro-1*H*-isoindol-1-one (3a)

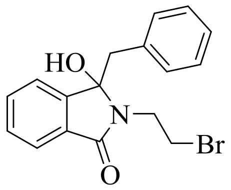

Appearance: colorless crystalline solid.

Melting point: 148°C.

IR (film): $\tilde{\nu}$ (cm^{-1}) = 3310 br, 3123, 1700, 1611, 1320 and 632.

^{1}H-NMR (300 MHz, $CDCl_3$): δ (ppm): 2.70 (br s, 1 H, OH), 3.14 (d, J = 15 Hz, 1 H, CH_2Ph), 3.52 (d, J = 15 Hz, 1 H, CH_2Ph), 3.61-3.82 (m, 3 H, CH_2Br, NCH_2), 4.11-4.17 (m, 1 H, NCH_2), 6.89 (d, J = 9 Hz, 2 H, CH), 7.11-7.17 (m, 3 H, CH), 7.20-7.23 (d, J = 9 Hz, 1 H, CH), 7.43-7.58 (m, 2 H, CH), 7.62 (d, J = 9 Hz, 1 H, CH).

^{13}C-NMR (75 MHz, $CDCl_3$): δ (ppm): 29.5 (t, 1 C, CH_2Br), 41.4 (t, 1 C, CH_2Ph), 43.3 (t, 1 C, NCH_2), 91.0 (s, 1 C, COH), 122.9 (d, 1 C, CH), 123.4 (d, 1 C, CH), 127.3 (d, 1 C, CH), 128.2 (d, 2 C, CH), 129.9 (d, 1 C, CH), 130.2 (d, 2 C, CH), 130.9 (d, 1 C, Cq), 132.3 (d, 1 C, CH), 134.3 (s, 1 C, Cq), 146.1 (s, 1 C, Cq), 167.0 (s, 1 C, C=O).

HRMS (ESI/MeOH): *m/z*: calcd for $C_{17}H_{16}O_2NBr(M+H)^+$: 346.0437 $(M+Na)^+$ 368.0257, found: 368.0261 ± 1 ppm.

2-(2-Bromoethyl)-3-(4-fluorobenzyl)-3-hydroxy-2,3-dihydro-1*H*-isoindol-1-one (3b)

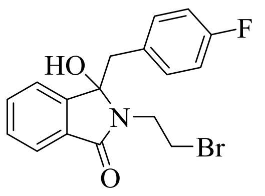

Appearance: colorless crystalline solid.

Melting point: 157°C.

IR (film): $\tilde{\nu}$ (cm^{-1}) = 3352 br, 3110, 1692, 1632, 1167 and 670.

^{1}H-NMR (300 MHz, $CDCl_3$): δ (ppm): 3.11 (d, J = 12 Hz, 1 H, CH_2Ar), 3.43 (d, J = 12 Hz, 1 H, CH_2Ar), 3.59-3.74 (m, 3 H, CH_2Br, NCH_2), 4.02-4.12 (m, 1 H, NCH_2), 7.03-7.08 (m, 2 H, CH), 7.14-7.25 (m, 4 H, CH), 7.50-7.57 (m, 2 H, CH), not observed (1 H, OH).

^{13}C-NMR (75 MHz, $CDCl_3$): δ (ppm): 33.1 (t, 1 C, CH_2Br), 39.3 (t, 1 C, NCH_2), 44.2 (t, 1 C, CH_2Ar), 90.0 (s, 1 C, COH), 120.4 (d, 1 C, CH), 122.1 (d, 1 C, CH), 129.2 (d, 2 C, CH), 131.2 (d, 1 C, CH), 132.7 (d, 2 C, CH), 133.3 (s, 1 C, Cq), 132.3 (d, 1 C, CH), 134.3 (s, 1 C, Cq), 145.0 (s, 1 C, Cq), 158.5 (s, 1 C, Cq), 166.6 (s, 1 C, C=O).

HRMS (ESI/MeOH): *m/z*: calcd for $C_{17}H_{15}O_2NBrF$ $(M+H)^+$: 364.0343, found: 364.0343 ± 0 ppm. calcd for $C_{17}H_{15}O_2NBrF$ $(M+Na)^+$: 386.0162, found: 386.0165 ± 1 ppm.

2-(2-Bromoethyl)-3-(4-chlorobenzyl)-3-hydroxy-2,3-dihydro-1*H*-isoindol-1-one (3c)

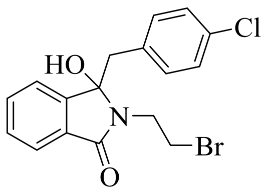

Appearance: pale yellow crystalline solid.

Melting point: 149-153°C.

IR (film): $\tilde{\nu}$ (cm^{-1}) = 3280 br, 3101, 1644, 1601, 1350 and 666.

^{1}H-NMR (300 MHz, $CDCl_3$): δ (ppm): 3.09 (d, J = 15 Hz, 1 H, CH_2Ar), 3.49 (d, J = 15 Hz, 1 H, CH_2Ar), 3.63-3.83 (m, 3 H, CH_2Br, NCH_2), 4.14-4.20 (m, 1 H, NCH_2), 6.83 (d, J = 9 Hz, 1 H, CH), 7.10-7.29 (m, 4 H, CH), 7.43-7.54 (m, 2 H, CH), 7.67 (d, J = 9 Hz, 1 H, CH), not observed (1 H, OH).

^{13}C-NMR (75 MHz, $CDCl_3$): δ (ppm): 32.6 (t, 1 C, CH_2Br), 40.0 (t, 1 C, NCH_2), 42.8 (t, 1 C, CH_2Ar), 90.0 (s, 1 C, COH), 119.3 (d, 1 C, CH), 121.9 (d, 1 C, CH), 122.2 (d, 1 C, CH), 122.6 (d, 1 C, CH), 123.8 (d, 1 C, CH), 128.2 (d, 1 C, CH), 130.2 (d, 1 C, CH), 132.3 (d, 1 C, CH), 134.5 (s, 1 C, Cq), 139.4 (s, 1 C, Cq), 144.8 (s, 1 C, Cq), 155.7 (s, 1 C, Cq), 166.3 (s, 1 C, C=O).

HRMS (ESI/MeOH): *m/z*: calcd for $C_{17}H_{15}NO_2BrCl$ $(M+Na)^+$: 401.9867, found: 401.9868 ± 1 ppm.

3-(4-Bromobenzyl)-2-(2-bromoethyl)-3-hydroxy-2,3-dihydro-1*H*-isoindol-1-one (3d)

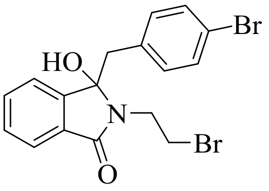

Appearance: pale yellow crystalline solid.

TLC (SiO_2, ethyl acetate/cyclohexane 4:6): R_f = 0.25.

Melting point: 158-160°C.

IR (film): $\tilde{\nu}$ (cm^{-1}) = 3537 br, 3127, 1665, 1471, 1364 and 654.

^{1}H-NMR (300 MHz, $CDCl_3$): δ (ppm): 3.05 (d, J = 15 Hz, 1 H, CH_2Ar), 3.47 (d, J = 15 Hz, 1 H, CH_2Ar), 3.61-3.77 (m, 3 H, CH_2Br, NCH_2), 4.09-4.17 (m, 1 H, NCH_2), 6.78 (d, J = 9 Hz, 2 H, CH), 6.97-7.18 (m, 2 H, CH), 7.29-7.54 (m, 3 H, CH), 7.64 (d, J = 9 Hz, 1 H, CH), not observed (1 H, OH).

^{13}C-NMR (75 MHz, acetone-d$_6$): δ (ppm): 30.4 (t, 1 C, CH_2Br), 40.7 (t, 1 C, NCH_2), 44.1 (t, 1 C, CH_2Ar), 91.5 (s, 1 C, COH), 121.3 (d, 1 C, CH), 122.6 (d, 1 C, CH), 125.2 (d, 2 C, CH), 129.0 (d, 1 C, CH), 130.1 (d, 1 C, CH), 132.5 (d, 2 C, CH), 135.3 (d, 1 C, Cq), 140.1 (s, 1 C, Cq), 143.4 (s, 1 C, Cq), 145.7 (s, 1 C, Cq), 166.6 (s, 1 C, C=O).

HRMS (ESI/MeOH): *m/z*: calcd for $C_{17}H_{15}NO_2Br_2$ $(M+Na)^+$: 445.9361, found: 445.9358 ± 1 ppm.

2-(2-Bromoethyl)-3-hydroxy-3-(4-methoxybenzyl)-2,3-dihydro-1*H*-isoindol-1-one (3e)

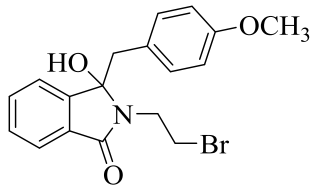

Appearance: pale yellow crystalline solid.

Melting point: 167-168°C.

IR (film): $\tilde{\nu}$ (cm^{-1}) = 3379 br, 3106, 1685, 1583, 1366, 1268 and 630.

^{1}H-NMR (300 MHz, CDCl$_3$): δ (ppm): 2.78 (br s, 1 H, OH), 3.13 (d, J = 15 Hz, 1 H, CH_2Ar), 3.49 (d, J = 15 Hz, 1 H, CH_2Ar), 3. 77 (s, 3 H, OCH_3), 3.72-3.82 (m, 3 H, CH_2Br, NCH_2), 4.15-4.24 (m, 1 H, NCH_2), 6.71 (d, J = 9 Hz, 2 H, CH), 6.84 (d, J = 9 Hz, 2 H, CH), 7.30 (m, 1 H, CH), 7.53 (m, 2 H, CH), 7.70 (d, J = 9 Hz, 1 H, CH).

^{13}C-NMR (75 MHz, acetone-d$_6$): δ (ppm): 29.7 (q, 1 C, OCH_3), 41.7 (t, 1 C, NCH_2), 42.5 (t, 1 C, CH_2Ar), 55.2 (t, 1 C, CH_2Ph), 91.9 (s, 1 C, COH), 113.8 (d, 2 C, CH), 123.1 (d, 1 C, CH), 123.7 (d, 1 C, CH), 127.8 (s, 1 C, Cq), 129.8 (d, 1 C, CH), 131.8 (d, 2 C, CH), 132.5 (d, 1 C, Cq), 135.1 (d, 1 C, CH), 147.8 (s, 1 C, Cq), 159.4 (s, 1 C, Cq), 167.0 (s, 1 C, C=O).

HRMS (ESI/MeOH): *m/z*: calcd for $C_{18}H_{18}NO_3Br$ $(M+Na)^+$: 398.0362, found: 398.0369 ± 2 ppm.

2-(2-Bromoethyl)-3-hydroxy-3-(4-methylbenzyl)-2,3-dihydro-1*H*-isoindol-1-one (3f)

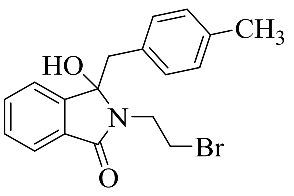

Appearance: pale yellow crystalline solid.

Melting point: 152°C.

IR (film): $\tilde{\nu}$ (cm^{-1}) = 3430 br, 3103, 1643, 1628, 1280 and 650.

^{1}H-NMR (300 MHz, $CDCl_3$): δ (ppm): 2.24 (s, 3 H, CH_3), 3.12 (d, J = 12 Hz, 1 H, CH_2Ar), 3.47 (d, J = 12 Hz, 1 H, CH_2Ar), 3.62-3.83 (m, 3 H, CH_2Br, NCH_2), 4.11-4.19 (m, 1 H, NCH_2), 6.74 (d, J = 6 Hz, 2 H, CH), 6.92 (d, J = 6 Hz, 2 H, CH), 7.28 (m, 1 H, CH), 7.41-7.54 (m, 2 H, CH), 7.64 (d, J = 6 Hz, 1 H, CH), not observed (1 H, OH).

^{13}C-NMR (75 MHz, acetone-d_6): δ (ppm): 20.1 (q, 1 C, CH_3), 31.3 (t, 1 C, CH_2Br), 38.2 (t, 1 C, NCH_2), 40.5 (t, 1 C, CH_2Ar), 90.2 (s, 1 C, COH), 120.9 (d, 1 C, CH), 121.0 (d, 1 C, CH), 123.8 (d, 2 C, CH), 126.9 (d, 1 C, CH), 129.1 (d, 1 C, CH), 130.5 (d, 2 C, CH), 138.9 (s, 1 C, Cq), 140.6 (s, 1 C, Cq), 141.7 (s, 1 C, Cq), 143.3 (s, 1 C, Cq), 166.9 (s, 1 C, C=O).

HRMS (ESI/MeOH): *m/z*: calcd for $C_{18}H_{18}NO_2Br$ $(M+Na)^+$: 382.0413, found: 382.0419 ± 2 ppm.

2-(2-Bromoethyl)-3-hydroxy-3-(3-methylbenzyl)-2,3-dihydro-1*H*-isoindol-1-one (3g)

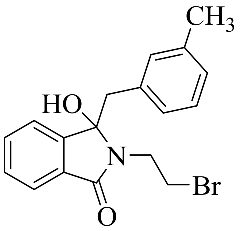

Appearance: pale yellow crystalline solid.

Melting point: 151-152°C.

^{1}H-NMR (300 MHz, $CDCl_3$): δ (ppm): 2.19 (s, 3 H, CH_3), 3.09 (d, J = 16 Hz, 1 H, CH_2Ar), 3.47 (d, J = 16 Hz, 1 H, CH_2Ar), 3.62-3.83 (m, 3 H, CH_2Br, NCH_2), 4.10-4.17 (m, 1 H, NCH_2), 6.65-6.69 (m, 2 H, CH), 7.02-7.04 (m, 2 H, CH), 7.22-7.25 (m, 1 H, CH), 7.43-7.57 (m, 2 H, CH), 7.62-7.65 (m, 1 H, CH), not observed (1 H, OH).

^{13}C-NMR (75 MHz, acetone-d_6): δ (ppm): 20.3 (q, 1 C, CH_3), 29.3 (t, 1 C, CH_2Br), 38.6 (t, 1 C, NCH_2), 42.1 (t, 1 C, CH_2Ar), 91.2 (s, 1 C, COH), 121.3 (d, 1 C, CH), 122.9 (d, 1 C, CH), 126.9 (d, 1 C, CH), 127.2 (d, 1 C, CH), 130.4 (d, 2 C, CH), 133.1 (s, 1 C, Cq), 134.7 (d, 2 C, CH), 139.0 (s, 1 C, Cq), 141.6 (s, 1 C, Cq), 143.9 (s, 1 C, Cq), 166.4 (s, 1 C, C=O).

HRMS (ESI/MeOH): *m/z*: calcd for $C_{18}H_{18}NO_2Br$ $(M+Na)^+$: 382.0413, found: 382.0419 ± 1 ppm.

2-(2-Bromoethyl)-3-hydroxy-3-(2-methylbenzyl)-2,3-dihydro-1*H*-isoindol-1-one (3h)

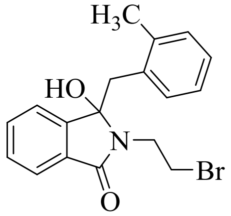

Appearance: colorless crystalline solid.

Melting point: 157°C.

^{1}H-NMR (300 MHz, $CDCl_3$): δ (ppm): 1.90 (s, 3 H, CH_3), 2.95 (d, J = 16 Hz, 1 H, CH_2Ar), 3.53 (d, J = 16 Hz, 1 H, CH_2Ar), 3.62-3.88 (m, 3 H, CH_2Br, NCH_2), 4.09-4.23 (m, 1 H, NCH_2), 6.69 (d, J = 9 Hz, 1 H, CH), 7.07-7.23 (m, 3 H, CH), 7.33-7.38 (m, 1 H, CH), 7.43-7.48 (m, 1 H, CH), 7.73-7.77 (m, 1 H, CH), 7.82-7.89 (m, 1 H, CH), not observed (1 H, OH).

^{13}C-NMR (75 MHz, acetone-d$_6$): δ (ppm): 19.7 (q, 1 C, CH_3), 30.9 (t, 1 C, CH_2Br), 41.2 (t, 1 C, NCH_2), 46.3 (t, 1 C, CH_2Ar), 91.7 (s, 1 C, COH), 119.1 (d, 1 C, CH), 121.9 (d, 1 C, CH), 122.2 (d, 1 C, CH), 122.8 (d, 1 C, CH), 128.0 (d, 1 C, CH), 130.0 (d, 1 C, CH), 132.2 (d, 1 C, CH), 134.0 (d, 1 C, CH), 135.5 (d, 1 C, Cq), 140.1 (s, 1 C, Cq), 143.4 (s, 1 C, Cq), 144.3 (s, 1 C, Cq), 167.1 (s, 1 C, C=O).

HRMS (ESI/MeOH): *m/z*: calcd for $C_{18}H_{18}NO_2Br$ $(M+Na)^+$: 382.0413, found: 382.0419 ± 1 ppm.

4-((2-(2-Bromoethyl)-1-hydroxy-3-oxoisoindolin-1-yl)methyl)phenyl acetate (3i)

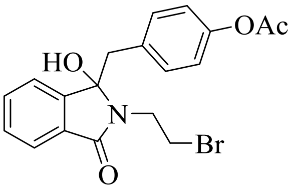

Appearance: yellow oil.

TLC (SiO_2, ethyl acetate/n-hexane 1:1): R_f = 0.46.

IR (film): $\tilde{\nu}$ (cm^{-1}) = 3282 br, 1762, 1684, 1415, 1191, 1068, 908, 697 and 618.

^{1}H-NMR (300 MHz, $CDCl_3$): δ (ppm): 2.05 (s, 3 H, CH_3), 3.25 (d, J = 14 Hz, 1 H, CH_2Ar), 3.44 (d, J = 14 Hz, 1 H, CH_2Ar), 3.54 (m, 2 H, CH_2Br), 5.67 (br s, 1 H, OH), 3.73 (m, 1 H, NCH_2), 3.94 (m, 1 H, NCH_2), 6.72 (d, J = 9 Hz, 2 H, CH), 6.83 (d, J = 9 Hz, 2 H, CH), 7.33 (m, 3 H, CH), 7.44 (m, 1 H, CH).

^{13}C-NMR (75 MHz, acetone-d$_6$): δ (ppm): 21.0 (q, 1 C, CH_3), 29.7 (t, 1 C, CH_2Br), 41.8 (t, 1 C, NCH_2), 42.8 (t, 1 C, CH_2Ar), 91.8 (s, 1 C, COH), 121.9 (d, 2 C, CH), 123.3 (s, 1 C, Cq), 130.2 (d, 2 C, CH), 131.9 (d, 2 C, CH), 132.2 (d, 1 C, CH), 132.7 (s, 1 C, Cq), 133.5 (s, 1 C, Cq), 147.7 (s, 1 C, Cq), 150.4 (d, 1 C, CH), 167.2 (s, 1 C, CO_2), 169.6 (s, 1 C, C=O).

3-Benzyl-2-(3-bromopropyl)-3-hydroxy-2,3-dihydro-1*H*-isoindol-1-one (3j)

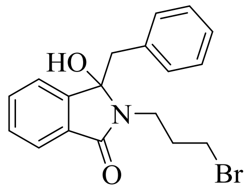

Appearance: Colorless crystalline solid.

Melting point: 160°C.

IR (film): $\tilde{\nu}$ (cm^{-1}) = 3260 br, 3150, 1680, 1650, 1300 and 685.

^{1}H-NMR (300 MHz, $CDCl_3$): δ (ppm): 2.26-2.45 (m, 2 H, CH_2), 2.80 (br s, 1 H, OH), 3.13 (d, J = 12 Hz, 1 H, CH_2Ph), 3.48-3.58 (m, 4 H, CH_2Ph, CH_2Br, NCH_2), 3.78-3.87 (m, 1 H, NCH_2), 6.92 (d, J = 9 Hz, 2 H, CH), 7.19-7.26 (m, 4 H, CH), 7.41-7.47 (m, 2 H, CH), 7.62 (d, J = 9 Hz, 1 H, CH).

^{13}C-NMR (75 MHz, $CDCl_3$): δ (ppm): 32.6 (t, 1 C, CH_2), 33.6 (t, 1 C, CH_2Br), 39.0 (t, 1 C, NCH_2), 43.5 (t, 1 C, CH_2Ph), 90.1 (s, 1 C, COH), 123.0 (d, 1 C, CH), 123.8 (d, 1 C, CH), 127.4 (d, 1 C, CH), 128.5 (s, 2 C, CH), 130.0 (s, 1 C, Cq), 131.0 (d, 2 C, CH), 132.3 (d, 1 C, CH), 132.7 (d, 1 C, CH), 136.2 (s, 1 C, Cq), 147.8 (s, 1 C, Cq), 167.2 (s, 1 C, C=O).

HRMS (ESI/MeOH): *m/z*: calcd for $C_{18}H_{18}NO_2Br$ $(M+Na)^+$: 382.0413, found: 382.0435 ± 6 ppm.

2-(3-Bromopropyl)-3-(4-fluorobenzyl)-3-hydroxy-2,3-dihydro-1*H*-isoindol-1-one (3k)

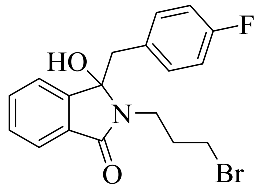

Appearance: yellowish solid.

Melting point: 161°C.

IR (film): $\tilde{\nu}$ (cm^{-1}) = 3382 br, 3110, 1690, 1660, 1146 and 680.

^{1}H-NMR (300 MHz, $CDCl_3$): δ (ppm): 2.16-2.44 (m, 2 H, CH_2), 3.09 (d, J = 12 Hz, 1 H, CH_2Ar), 3.47-3.54 (m, 4 H, CH_2Ar, CH_2Br, NCH_2), 3.76-3.83 (m, 1 H, NCH_2), 6.80-6.89 (m, 4 H, CH), 7.11 (d, J = 9 Hz, 1 H, CH), 7.42-7.47 (m, 2 H, CH), 7.61 (d, J = 9 Hz, 1 H, CH), not observed (1 H, OH).

^{13}C-NMR (75 MHz, $CDCl_3$): δ (ppm): 30.0 (t, 1 C, CH_2), 34.8 (t, 1 C, CH_2Br), 40.2 (t, 1 C, NCH_2), 45.5 (t, 1 C, CH_2Ar), 93.1 (s, 1 C, COH), 120.1 (d, 1 C, CH), 122.7 (d, 1 C, CH), 124.7 (d, 1 C, CH), 128.2 (d, 2 C, CH), 130.7 (s, 1 C, Cq), 131.3 (d, 2 C, CH), 132.9 (d, 1 C, CH), 135.7 (s, 1 C, Cq), 136.2 (s, 1 C, Cq), 158.1 (s, 1 C, Cq), 167.0 (s, 1 C, C=O).

HRMS (ESI/MeOH): *m/z*: calcd for $C_{18}H_{17}O_2NFBr$ $(M+Na)^+$: 400.0319, found: 400.0340 ± 5 ppm.

2-(3-Bromopropyl)-3-(4-chlorobenzyl)-3-hydroxy-2,3-dihydro-1*H*-isoindol-1-one (3l)

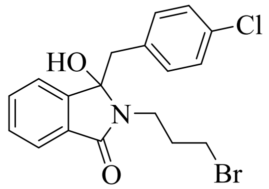

Appearance: yellowish solid.

Melting point: 158-160°C.

IR (film): $\tilde{\nu}$ (cm^{-1}) = 3380 br, 3091, 1683, 16027, 1338 and 671.

^{1}H-NMR (300 MHz, $CDCl_3$): δ (ppm): 2.24-2.43 (m, 2 H, CH_2), 3.05 (d, J = 12 Hz, 1 H, CH_2Ar), 3.41-3.54 (m, 4 H, CH_2Ar, CH_2Br, NCH_2), 3.73-3.82 (m, 1 H, NCH_2), 6.85 (d, J = 6 Hz, 2 H, CH), 7.09-7.13 (m, 3 H, CH), 7.40-7.46 (m, 2 H, CH), 7.58 (d, J = 6 Hz, 1 H, CH), not observed (1 H, OH).

^{13}C-NMR (75 MHz, $CDCl_3$): δ (ppm): 29.7 (t, 1 C, CH_2), 33.2 (t, 1 C, CH_2Br), 41.6 (t, 1 C, NCH_2), 44.9 (t, 1 C, CH_2Ar), 93.1 (s, 1 C, COH), 119.6 (d, 1 C, CH), 120.1 (d, 1

C, CH), 122.4 (d, 1 C, CH), 123.7 (d, 1 C, CH), 125.3 (d, 1 C, CH), 129.3 (s, 1 C, Cq), 130.5 (s, 1 C, CH), 131.4 (s, 1 C, CH), 132.0 (s, 1 C, CH), 134.3 (s, 1 C, Cq), 135.9 (s, 1 C, Cq), 140.4 (s, 1 C, Cq), 167.3 (s, 1 C, C=O).

HRMS (ESI/MeOH): *m/z*: calcd for $C_{18}H_{16}BrClNO_2$ $(M+Na)^+$: 416.0024, found: 416.0027 ± 1 ppm.

2-(3-Bromopropyl)-3-(4-bromobenzyl)-3-hydroxy-2,3-dihydro-1*H*-isoindol-1-one (3m)

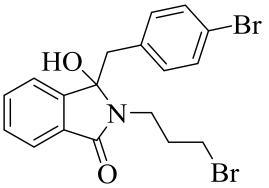

Appearance: slightly off-white crystalline solid.

Melting point: 159°C.

IR (film): $\tilde{\nu}$ (cm^{-1}) = 3550 br, 3120, 1685, 1401, 1324 and 683.

^{1}H-NMR (300 MHz, $CDCl_3$): δ (ppm): 2.27-2.40 (m, 2 H, CH_2), 3.05 (d, J = 15 Hz, 1 H, CH_2Ar), 3.44-3.53 (m, 4 H, CH_2Ar, CH_2Br, NCH_2), 3.76-3.84 (m, 1 H, NCH_2), 6.80 (d, J = 9 Hz, 2 H, CH), 7.09-7.12 (m, 1 H, CH), 7.27-7.29 (m, 2 H, CH), 7.41-7.48 (d, J = 9 Hz, 2 H, CH), 7.62 (d, J = 9 Hz, 1 H, CH), not observed (1 H, OH).

^{13}C-NMR (75 MHz, $CDCl_3$): δ (ppm): 30.1 (t, 1 C, CH_2), 34.7 (t, 1 C, CH_2Br), 40.9 (t, 1 C, NCH_2), 45.0 (t, 1 C, CH_2Ar), 90.5 (s, 1 C, COH), 120.5 (d, 2 C, CH), 122.4 (d, 1 C, CH), 123.7 (d, 1 C, CH), 125.3 (d, 1 C, CH), 129.3 (d, 1 C, CH), 130.5 (s, 1 C, Cq), 131.4 (d, 1 C, CH), 132.0 (d, 1 C, CH), 134.3 (s, 1 C, Cq), 135.9 (s, 1 C, Cq), 140.4 (s, 1 C, Cq), 166.2 (s, 1 C, C=O).

HRMS (ESI/MeOH): *m/z*: calcd for $C_{18}H_{17}NO_2Br_2$ $(M+Na)^+$: 459.9518, found: 459.9548 ± 7 ppm.

2-(3-Bromopropyl)-3-hydroxy-3-(4-methoxybenzyl)-2,3-dihydro-1*H*-isoindol-1-one (3n)

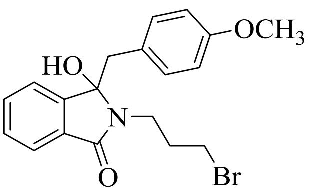

Appearance: colorless crystalline solid.

Melting point: 167-168°C.

IR (film): $\tilde{\nu}$ (cm^{-1}) = 3380 br, 3123, 1670, 1597, 1328, 1250 and 647.

^{1}H-NMR (300 MHz, $CDCl_3$): δ (ppm): 2.37-2.46 (m, 2 H, CH_2), 3.32 (d, J = 15 Hz, 1 H, CH_2Ar), 3.54 (d, J = 15 Hz, 1 H, CH_2Ar), 3.64 (t, J = 6 Hz, 2 H, CH_2Br), 3.71 (s, 3 H, OCH_3), 3.79 (m, 1 H, NCH_2), 3.83-3.92 (m, 1 H, NCH_2), 5.70 (br s, 1 H, OH), 6.68 (d, J = 9 Hz, 2 H, CH), 6.84-6.89 (m, 2 H, CH), 7.17 (d, J = 9 Hz, 1 H, CH), 7.45-7.50 (m, 3 H, CH).

^{13}C-NMR (75 MHz, acetone-d_6): δ (ppm): 31.4 (q, 1 C, OCH_3), 31.9 (t, 1 C, CH_2), 36.8 (t, 1 C, CH_2Br), 38.6 (t, 1 C, NCH_2), 55.6 (t, 1 C, CH_2Ar), 89.2 (s, 1 C, COH), 114.5 (d, 1 C, CH), 123.5 (d, 1 C, CH), 124.3 (d, 1 C, CH), 128.1 (d, 2 C, CH), 129.6 (s, 1 C, Cq), 130.1 (d, 2 C, CH), 132.4 (s, 1 C, Cq), 133.1 (d, 1 C, CH), 134.9 (s, 1 C, Cq), 136.0 (s, 1 C, Cq), 166.5 (s, 1 C, C=O).

HRMS (ESI/MeOH): *m/z*: calcd for $C_{19}H_{20}NO_3Br$ $(M+Na)^+$: 412.0519, found: 412.0512 ± 2 ppm.

2-(3-Bromopropyl)-3-hydroxy-3-(4-methylbenzyl)-2,3-dihydro-1*H*-isoindol-1-one (3o)

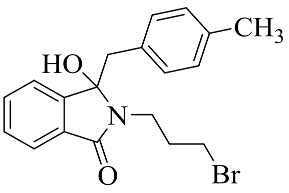

Appearance: colorless crystalline solid.

Melting point: 156-157°C.

IR (film): $\tilde{\nu}$ (cm^{-1}) = 3442 br, 3232, 1680, 1600, 1272 and 664.

^{1}H-NMR (300 MHz, $CDCl_3$): δ (ppm): 2.25 (s, 3 H, CH_3), 2.28-2.43 (m, 3 H, CH_2, CH_2Ar), 3.11 (d, J = 12 Hz, 1 H, CH_2Ar), 3.48-3.57 (m, 4 H, CH_2Br, NCH_2), 6.78 (d, 2 H, J = 9 Hz, CH), 6.94 (d, 2 H, J = 9 Hz, CH), 7.18-7.21 (m, 1 H, CH), 7.39-7.50 (m, 2 H, CH), 7.62 (d, J = 9 Hz, 1 H, CH), not observed (1 H, OH).

^{13}C-NMR (75 MHz, $CDCl_3$): δ (ppm): 20.6 (q, 1 C, CH_3), 30.2 (t, 1 C, CH_2), 34.7 (t, 1 C, CH_2Br), 40.1 (t, 1 C, NCH_2), 45.0 (t, 1 C, CH_2Ar), 90.3 (s, 1 C, COH), 121.3 (d, 2 C, CH), 122.7 (d, 1 C, CH), 125.0 (d, 2 C, CH), 129.0 (s, 1 C, Cq), 130.8 (s, 1 C, CH), 131.3 (s, 1 C, CH), 132.0 (s, 1 C, CH), 134.3 (s, 1 C, Cq), 135.9 (s, 1 C, Cq), 140.4 (s, 1 C, Cq), 166.5 (s, 1 C, C=O).

HRMS (ESI/MeOH): *m/z*: calcd for $C_{19}H_{20}O_2NBr$ $(M+Na)^+$: 396.0570, found: 396.0568 ± 1 ppm.

2-(3-Bromopropyl)-3-hydroxy-3-(3-methylbenzyl)-2,3-dihydro-1*H*-isoindol-1-one (3p)

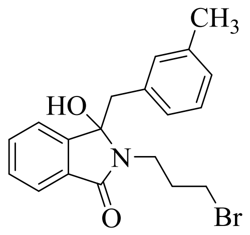

Appearance: yellowish powder.

Melting point: 156-158°C.

IR (film): $\tilde{\nu}$ (cm^{-1}) = 3453 br, 3156, 1684, 1645, 1297 and 662.

^{1}H-NMR (300 MHz, $CDCl_3$): δ (ppm): 1.91 (s, 3 H, CH_3), 2.32-2.51 (m, 3 H, CH_2Ar, CH_2Br), 2.91 (d, J = 15 Hz, 1 H, CH_2Ar), 3.50-3.62 (m, 4 H, CH_2Br, NCH_2), 6.61 (d, J

= 9 Hz, 1 H, CH), 7.08-7.20 (m, 3 H, CH), 7.32-7.46 (m, 3 H, CH), 7.73 (d, J = 9 Hz, 1 H, CH), not observed (1 H, OH).

^{13}C-NMR (75 MHz, $CDCl_3$): δ (ppm): 20.3 (q, 1 C, CH_3), 29.7 (t, 1 C, CH_2), 33.8 (t, 1 C, CH_2Br), 42.0 (t, 1 C, NCH_2), 44.9 (t, 1 C, CH_2Ar), 91.0 (s, 1 C, COH), 121.1 (d, 1 C, CH), 122.4 (d, 1 C, CH), 123.0 (d, 1 C, CH), 123.7 (d, 1 C, CH), 128.1 (d, 1 C, CH), 130.1 (s, 1 C, Cq), 131.5 (s, 1 C, CH), 131.9 (s, 1 C, CH), 132.7 (s, 1 C, CH), 134.0 (s, 1 C, Cq), 135.3 (s, 1 C, Cq), 139.7 (s, 1 C, Cq), 166.6 (s, 1 C, C=O).

HRMS (ESI/MeOH): *m/z*: calcd for $C_{19}H_{20}O_2NBr$ $(M+Na)^+$: 396.0570, found: 396.0585 ± 4 ppm.

2-(3-Bromopropyl)-3-hydroxy-3-(2-methylbenzyl)-2,3-dihydro-1*H*-isoindol-1-one (3q)

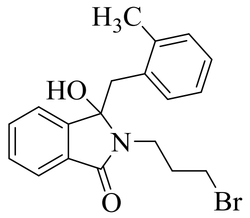

Appearance: pale yellow crystalline solid.

Melting point: 155°C.

IR (film): $\tilde{\nu}$ (cm^{-1}) = 3396 br, 3145, 1689, 1630, 1207 and 692.

^{1}H-NMR (300 MHz, $CDCl_3$): δ (ppm): 2.24 (s, 3 H, CH_3), 2.32-2.42 (m, 2 H, CH_2Br), 3.11 (d, J = 15 Hz, 1 H, CH_2Ar), 3.50-3.57 (m, 2 H, CH_2Br, CH_2Ar), 3.76-3.86 (m, 1 H, NCH_2), 4.10-4.17 (m, 2 H, CH_2Br, NCH_2), 6.72-6.77 (m, 2 H, CH), 7.00-7.06 (m, 2 H, CH), 7.17-7.24 (m, 1 H, CH), 7.44-7.50 (m, 2 H, CH), 7.58-7.62 (m, 1 H, CH), not observed (1 H, OH).

^{13}C-NMR (75 MHz, $CDCl_3$): δ (ppm): 20.1 (q, 1 C, CH_3), 29.7 (t, 1 C, CH_2), 33.2 (t, 1 C, CH_2Br), 41.6 (t, 1 C, NCH_2), 44.9 (t, 1 C, CH_2Ar), 90.1 (s, 1 C, COH), 120.1 (d, 1 C, CH), 121.9 (d, 1 C, CH), 123.7 (d, 1 C, CH), 124.0 (d, 1 C, CH), 126.0 (d, 1 C,

CH), 129.1 (s, 1 C, Cq), 130.7 (d, 1 C, CH), 131.4 (d, 1 C, CH), 132.3 (d, 1 C, CH), 135.8 (s, 2 C, Cq), 140.0 (s, 1 C, Cq), 167.0 (s, 1 C, C=O).

HRMS (ESI/MeOH): *m/z*: calcd for $C_{19}H_{20}O_2NBr$ $(M+Na)^+$: 396.0570, found: 396.0570 ± 0 ppm.

(1-Benzyl-1-hydroxy-3-oxoisoindolin-2-yl)methyl 2-phenylacetate (5)

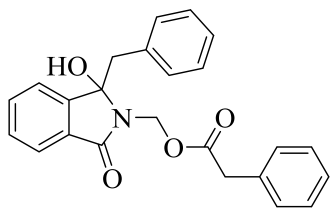

Appearance: colorless oil.

TLC (SiO$_2$, ethyl acetate/n-hexane 1:1): R_f = 0.42.

IR (film): $\tilde{\nu}$ (cm^{-1}) = 2724 br, 1727, 1678, 1601, 1513, 1155, 967, 722 and 665.

^{1}H-NMR (300 MHz, acetone-d$_6$): δ (ppm): 3.24 (d, J = 14 Hz, 1 H, CH_2Ph), 3.54 (d, J = 14 Hz, 1 H, CH_2Ph), 3.72 (s, 2 H, O_2CCH_2Ph), 5.69 (d, J = 11 Hz, 1 H, NCH_2), 5.73 (s, 1 H, OH), 5.84 (d, J = 11 Hz, 1 H, NCH_2), 7.11 (m, 5 H, CH), 7.28 (m, 7 H, CH), 7.56 (m, 2 H, CH).

^{13}C-NMR (75 MHz, acetone-d$_6$): δ (ppm): 40.9 (t, 1 C, CH_2Ph), 43.9 (t, 1 C, CH_2Ph), 64.1 (t, 1 C, NCH_2), 90.7 (d, 1 C, COH), 123.1 (d, 1 C, CH), 123.8 (d, 1 C, CH), 126.8 (d, 1 C, CH), 127.1 (d, 1 C, CH), 127.9 (d, 1 C, CH), 128.6 (d, 2 C, CH), 129.6 (d, 2 C, CH), 129.7 (d, 2 C, CH), 130.6 (d, 1 C, CH), 130.7 (d, 1 C, CH), 132.6 (d, 2 C, CH), 134.6 (s, 1 C, Cq), 135.6 (s. 1 C, Cq), 147.3 (s, 1 C, Cq), 167.0 (s, 1 C, C=O), 171.0 (s, 1 C, C=O).

9b-Benzyl-2,3-dihydrooxazolo[2,3-a]isoindol-5(9b*H*)-one (4) [3]

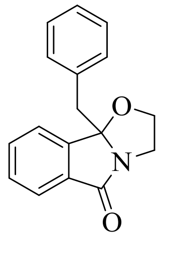

Appearance: yellowish oil.

^{1}H-NMR (300 MHz, acetone-d$_6$): δ (ppm): 3.14 (m, 1 H, NCH_2), 3.30 (d, J = 14 Hz, 1 H, CH_2Ph), 3.44 (d, J = 14 Hz, 1 H, CH_2Ph), 3.98 (m, 2 H, CH_2O), 4.17 (m, 1 H, NCH_2), 7.12-7.26 (m, 5 H, CH), 7.47-7.72 (m, 4 H, CH).

^{13}C-NMR (75 MHz, acetone-d$_6$): δ (ppm): 43.4 (t, 1 C, CH_2Ph), 44.5 (t, 1 C, NCH_2), 71.3 (t, 1 C, CH_2O), 102.4 (s, 1 C, CqO), 125.2 (d, 1 C, CH), 125.3 (d, 1 C, CH), 128.5 (d, 1 C, CH), 129.7 (d, 2 C, CH), 131.9 (d, 1 C, CH), 132.5 (d, 2 C, CH), 134.0 (s, 1 C, Cq), 134.7 (d, 1 C, CH), 137.6 (s, 1 C, Cq), 148.7 (s, 1 C, Cq), 174.9 (s, 1 C, C=O).

Dehydrations

3-Benzylidene-2-(2-bromoethyl)-2,3-dihydro-1*H*-isoindol-1-one (7a)

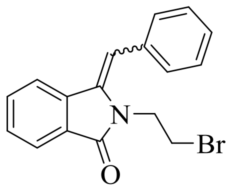

Appearance: yellowish powder.

TLC (SiO_2, ethyl acetate/n-hexane 1:1): R_f = 0.89.

Melting point: 102°C.

IR (film): $\tilde{\nu}$ (cm^{-1}) = 3140, 1658, 1620, 1580, 1301, 832 and 689.

^{1}H-NMR (mixture of stereoisomers, 300 MHz, $CDCl_3$): Main *E*-isomer: δ (ppm): 3.67 (t, J = 6 Hz, 2 H, CH_2Br), 4.34 (t, J = 6 Hz, 2 H, NCH_2), 6.65 (s, 1 H, CH.), 7.30-7.50 (m, 8 H, CH), 7.90 (d, J = 9 Hz, 1 H, CH). **Minor *Z*-isomer:** characteristic peaks only. δ (ppm): 3.15 (t, J = 6 Hz, 2 H, CH_2Br), 4.14 (t, J = 6 Hz, 2 H, NCH_2), 6.85 (s, 1 H, CH.).

^{13}C-NMR (mixture of stereoisomers, 75 MHz, $CDCl_3$): Main *E*-isomer: δ (ppm): 27.7 (t, 1 C, CH_2Br), 41.1 (t, 1 C, NCH_2), 110.4 (d, 1 C, CH), 123.3 (s, 1 C, Cq), 123.4 (d, 2 C, CH), 128.9 (d, 2 C, CH), 129.3 (d, 1 C, CH), 129.6 (d, 1 C, CH), 129.6 (d, 1

C, CH), 129.9 (d, 1 C, CH), 132.0 (s, 1 C, Cq), 134.8 (d, 1 C, CH), 135.7 (d, 1 C, Cq), 135.8 (s, 1 C, Cq), 166.6 (s, 1 C, C=O). **Minor *Z*-isomer:** quantity too low to assign peaks.

HRMS (mixture of stereoisomers, ESI/MeOH): *m/z*: calcd for $C_{17}H_{14}ONBr$ $(M+Na)^+$: 350.0151, found: 350.0149 ± 1 ppm.

2-(2-Bromoethyl)-3-(4-fluorobenzylidene)-2,3-dihydro-1*H*-isoindol-1-one (7b)

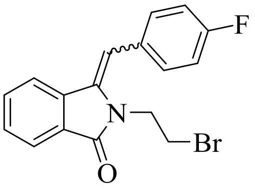

Appearance: yellow oil.

IR (film): $\tilde{\nu}$ (cm^{-1}) = 3125, 1692, 1632, 1546, 1167, 830, 670.

^{1}H-NMR (mixture of stereoisomers, 300 MHz, $CDCl_3$): Main *E*-isomer: δ (ppm): 3.53 (t, J = 6 Hz, 2 H, CH_2Br), 4.37 (t, J = 6 Hz, 2 H, NCH_2), 6.60 (s, 1 H, CH), 7.11-7.15 (m, 2 H, CH), 7.45-7.50 (m, 2 H, CH), 7.90 (m, 4 H, CH). **Minor *Z*-isomer:** quantity too low to assign peaks.

^{13}C-NMR (mixture of stereoisomers, 75 MHz, $CDCl_3$): Main *E*-isomer: δ (ppm): 33.0 (t, 1 C, CH_2Br), 40.4 (t, 1 C, NCH_2), 107.0 (d, 1 C, CH), 114.3 (d, 2 C, CH), 123.5 (d, 1 C, CH), 123.7 (s, 1 C, Cq), 127.0 (s, 1 C, Cq), 128.2 (d, 1 C, CH), 129.4 (d, 1 C, CH), 130.9 (d, 2 C, CH), 131.9 (s, 1 C, Cq), 134.4 (d, 1 C, CH), 134.3 (s, 1 C, Cq), 158.6 (s, 1 C, Cq), 166.6 (s, 1 C, C=O). **Minor *Z*-isomer:** quantity too low to assign peaks.

HRMS (mixture of stereoisomers, ESI/MeOH): *m/z*: calcd for $C_{17}H_{13}ONBrF$ $(M+Na)^+$: 368.0057, found: 368.0059 ± 1 ppm.

2-(2-Bromoethyl)-3-(4-chlorobenzylidene)-2,3-dihydro-1*H*-isoindol-1-one (7c)

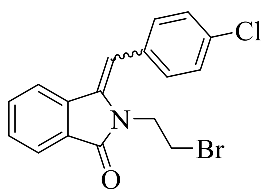

Appearance: crystalline dark yellowish solid.

Melting point: 120°C.

IR (film): $\tilde{\nu}$ (cm^{-1}) = 3112, 1644, 1601, 1558, 1350, 845 and 666.

^{1}H-NMR (mixture of stereoisomers, 300 MHz, $CDCl_3$): Main *E*-isomer: δ (ppm): 3.62 (t, J = 9 Hz, 2 H, CH_2Br), 4.28 (t, J = 9 Hz, 2 H, NCH_2), 6.53 (s, 1 H, CH), 7.24-7.30 (m, 2 H, CH), 7.36-7.49 (m, 5 H, CH), 7.84-7.87 (m, 1 H, CH). **Minor *Z*-isomer:** quantity too low to assign peaks.

^{13}C-NMR (mixture of stereoisomers, 75 MHz, $CDCl_3$): Main *E*-isomer: δ (ppm): 32.8 (t, 1 C, CH_2Br), 40.0 (t, 1 C, NCH_2), 106.9 (d, 1 C, CH), 117.0 (d, 2 C, CH), 123.0 (d, 1 C, CH), 124.9 (s, 1 C, Cq), 127.0 (s, 1 C, Cq), 128.7 (d, 1 C, CH), 129.2 (d, 1 C, CH), 130.0 (d, 2 C, CH), 131.5 (s, 1 C, Cq), 133.8 (d, 1 C, CH), 134.0 (s, 1 C, Cq), 155.7 (s, 1 C, Cq), 166.0 (s, 1 C, C=O). **Minor *Z*-isomer:** quantity too low to assign peaks.

HRMS (mixture of stereoisomers, ESI/MeOH): *m/z*: calcd for $C_{17}H_{13}ONBrCl$ $(M+Na)^+$: 383.9761, found: 383.9762 ± 1 ppm.

3-(4-Bromobenzylidene)-2-(2-bromoethyl)-2,3-dihydro-1*H*-isoindol-1-one (7d)

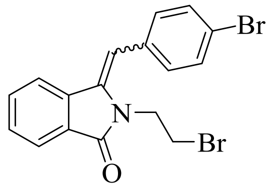

Appearance: yellow oil.

IR (film): $\tilde{\nu}$ (cm^{-1}) = 3138, 1665, 1471, 1572, 1364, 841 and 654.

^{1}H-NMR (mixture of stereoisomers, 300 MHz, $CDCl_3$): Main *E*-isomer: δ (ppm): 3.63 (t, J = 9 Hz, 2 H, CH_2Br), 4.29 (t, J = 9 Hz, 2 H, NCH_2), 6.53 (s, 1 H, CH), 7.11-7.17 (dd, J = 9, 3 Hz, 2 H, CH), 7.32-7.48 (m, 5 H, CH), 7.86 (d, J = 9 Hz, 1 H, CH). **Minor *Z*-isomer:** quantity too low to assign peaks.

^{13}C-NMR (mixture of stereoisomers, 75 MHz, $CDCl_3$): Main *E*-isomer: δ (ppm): 29.9 (t, 1 C, CH_2Br), 40.4 (t, 1 C, NCH_2), 106.4 (d, 1 C, CH), 114.7 (d, 1 C, CH), 115.0 (d, 1 C, CH), 123.2 (d, 1 C, CH), 123.3 (s, 1 C, Cq), 127.8 (s, 1 C, Cq), 128.9 (d, 1 C, CH), 129.6 (d, 1 C, CH), 130.3 (d, 2 C, CH), 131.7 (s, 1 C, Cq), 134.7 (d, 1 C, CH), 135.2 (s, 1 C, Cq), 145.3 (s, 1 C, Cq), 167.2 (s, 1 C, C=O). **Minor *Z*-isomer:** quantity too low to assign peaks.

HRMS (mixture of stereoisomers, ESI/MeOH): *m/z*: calcd for $C_{17}H_{14}ONBr_2$ $(M+Na)^+$: 427.9256, found: 427.9255 ± 1 ppm.

2-(2-Bromoethyl)-3-(4-methoxybenzylidene)-2,3-dihydro-1*H*-isoindol-1-one (7e)

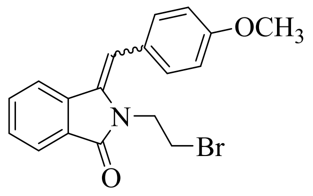

Appearance: yellow oil.

TLC (SiO_2, ethyl acetate/n-hexane 1:1): R_f = 0.74.

IR (film): $\tilde{\nu}$ (cm^{-1}) = 3126, 1685, 1583, 1581, 1366, 1268, 826 and 630.

^{1}H-NMR (mixture of stereoisomers, 300 MHz, $CDCl_3$): Main *E*-isomer: δ (ppm): 3.66 (t, J = 9 Hz, 2 H, CH_2Br), 3.93 (s, 3 H, OCH_3), 4.33 (t, J = 9 Hz, 2 H, NCH_2), 6.60 (s, 1 H, CH), 7.02 (d, J = 9 Hz, 2 H, CH), 7.40-7.43 (m, 5 H, CH), 7.90 (d, J = 9 Hz, 1 H, CH). **Minor *Z*-isomer:** quantity too low to assign peaks.

^{13}C-NMR (mixture of stereoisomers, 75 MHz, $CDCl_3$): Main *E*-isomer: δ (ppm): 27.8 (t, 1 C, CH_2Br), 42.1 (t, 1 C, NCH_2), 55.2 (q, 1 C, OCH_3), 107.0 (d, 1 C, CH), 114.3 (d, 2 C, CH), 123.5 (d, 1 C, CH), 123.7 (s, 1 C, Cq), 127.0 (s, 1 C, Cq), 128.2

(d, 1 C, CH), 129.4 (d, 1 C, CH), 130.9 (d, 2 C, CH), 131.9 (s, 1 C, Cq), 134.4 (d, 1 C, CH), 134.3 (s, 1 C, Cq), 159.6 (s, 1 C, Cq), 166.6 (s, 1 C, C=O). **Minor *Z*-isomer:** quantity too low to assign peaks.

HRMS (mixture of stereoisomers, ESI/MeOH): *m/z*: calcd for $C_{18}H_{16}O_2NBr$ $(M+Na)^+$: 380.0257, found: 380.0252 ± 1 ppm.

2-(2-Bromoethyl)-3-(4-methylbenzylidene)-2,3-dihydro-1*H*-isoindol-1-one (7f)

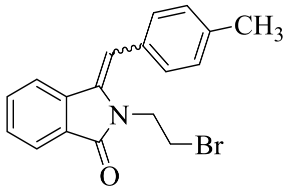

Appearance: yellow oil.

IR (film): $\tilde{\nu}$ (cm^{-1}) = 3101, 1643, 1628, 1550, 1278, 837 and 678.

^{1}H-NMR (mixture of stereoisomers, 300 MHz, $CDCl_3$): Main *E*-isomer: δ (ppm): 2.43 (s, 3 H, CH_3), 3.62 (t, J = 9 Hz, 2 H, CH_2Br), 4.29 (t, J = 9 Hz, 2 H, NCH_2), 6.59 (s, 1 H, CH), 7.33-7.46 (m, 7 H, CH), 7.85 (d, J = 6 Hz, 1 H, CH). **Minor *Z*-isomer:** quantity too low to assign peaks.

^{13}C-NMR (mixture of stereoisomers, 75 MHz, $CDCl_3$): Main *E*-isomer: δ (ppm): 21.0 (q, 1 C, CH_3), 27.8 (t, 1 C, CH_2Br), 41.1 (t, 1 C, NCH_2), 106.9 (d, 1 C, CH), 115.9 (d, 1 C, CH), 120.2 (d, 1 C, CH), 123.6 (d, 1 C, CH), 123.6 (s, 1 C, Cq), 125.9 (d, 1 C, CH), 126.9 (s, 1 C, Cq), 129.1 (d, 1 C, CH), 129.8 (d, 1 C, CH), 130.2 (d, 1 C, CH), 131.4 (s, 1 C, Cq), 134.7 (d, 1 C, CH), 135.1 (s, 1 C, Cq), 138.4 (s, 1 C, Cq), 167.8 (s, 1 C, C=O). **Minor *Z*-isomer:** quantity too low to assign peaks.

HRMS (mixture of stereoisomers, ESI/MeOH): *m/z*: calcd for $C_{18}H_{16}NOBr$ $(M+Na)^+$: 364.0307, found: 364.0307 ± 1 ppm.

2-(2-Bromoethyl)-3-(3-methylbenzylidene)-2,3-dihydro-1*H*-isoindol-1-one (7g)

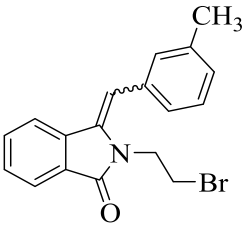

Appearance: yellow oil.

IR (film): $\tilde{\nu}$ (cm^{-1}) = 3129, 1632, 1601, 1565, 1282, 841 and 678.

^{1}H-NMR (mixture of stereoisomers, 300 MHz, $CDCl_3$): Main *E*-isomer: δ (ppm): 2.40 (s, 3 H, CH_3), 3.62 (t, J = 6 Hz, 2 H, CH_2Br), 4.12 (t, J = 6 Hz, 2 H, NCH_2), 6.60 (s, 1 H, CH), 7.13-7.23 (m, 2 H, CH), 7.30-7.36 (m, 2 H, CH), 7.40-7.46 (m, 1 H, CH), 7.73-7.77 (m, 1 H, CH), 7.83-7.89 (m, 2 H, CH). **Minor *Z*-isomer:** quantity too low to assign peaks.

^{13}C-NMR (mixture of stereoisomers, 75 MHz, $CDCl_3$): Main *E*-isomer: δ (ppm): 21.0 (q, 1 C, CH_3), 27.5 (t, 1 C, CH_2Br), 40.3 (t, 1 C, NCH_2), 110.4 (d, 1 C, CH), 114.5 (d, 1 C, CH), 120.3 (d, 1 C, CH), 122.6 (d, 1 C, CH), 123.6 (s, 1 C, Cq), 126.9 (s, 1 C, Cq), 129.1 (d, 1 C, CH), 129.8 (d, 1 C, CH), 130.5 (d, 1 C, CH), 130.9 (d, 1 C, CH), 131.2 (s, 1 C, Cq), 133.9 (d, 1 C, CH), 135.3 (s, 1 C, Cq), 138.7 (s, 1 C, Cq), 167.6 (s, 1 C, C=O). **Minor *Z*-isomer:** quantity too low to assign peaks.

HRMS (mixture of stereoisomers, ESI/MeOH): *m/z*: calcd for $C_{18}H_{16}NOBr$ $(M+Na)^+$: 364.0307, found: 364.0309 ± 1 ppm.

2-(2-Bromoethyl)-3-(2-methylbenzylidene)-2,3-dihydro-1*H*-isoindol-1-one (7h)

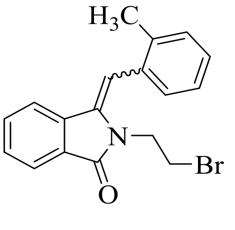

Appearance: yellow oil.

IR (film): $\tilde{\nu}$ (cm^{-1}) = 3130, 1633, 1600, 1572, 1260, 832 and 681.

^{1}H-NMR (mixture of stereoisomers, 300 MHz, $CDCl_3$): Main *E*-isomer: δ (ppm): 2.31 (s, 3 H, CH_3), 3.66 (t, J = 9 Hz, 2 H, CH_2Br), 4.12 (t, J = 9 Hz, 2 H, NCH_2), 6.56 (s, 1 H, CH), 6.96 (d, J = 6 Hz, 1 H, CH), 7.29-7.44 (m, 5 H, CH), 7.73-7.89 (m, 2 H, CH). **Minor *Z*-isomer:** quantity too low to assign peaks.

^{13}C-NMR (mixture of stereoisomers, 75 MHz, $CDCl_3$): Main *E*-isomer: δ (ppm): 21.3 (q, 1 C, CH_3), 27.3 (t, 1 C, CH_2Br), 40.0 (t, 1 C, NCH_2), 107.0 (d, 1 C, CH), 117.3 (d, 1 C, CH), 121.7 (d, 1 C, CH), 123.1 (s, 1 C, Cq), 123.8 (d, 1 C, CH), 126.9 (s, 1 C, Cq), 128.9 (d, 1 C, CH), 129.7 (d, 1 C, CH), 130.9 (d, 2 C, CH), 131.5 (s, 1 C, Cq), 134.3 (d, 1 C, CH), 135.1 (s, 1 C, Cq), 138.9 (s, 1 C, Cq), 167.5 (s, 1 C, C=O). **Minor *Z*-isomer:** quantity too low to assign peaks.

HRMS (mixture of stereoisomers, ESI/MeOH): *m/z*: calcd for $C_{18}H_{16}NOBr$ $(M+Na)^+$: 364.0307, found: 364.0305 ± 1 ppm.

4-((2-(2-Bromoethyl)-3-oxoisoindolin-1-ylidene)methyl)phenyl acetate (7i)

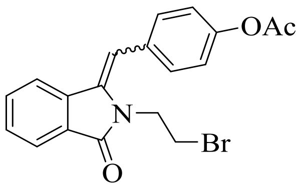

Appearance: yellow oil.

TLC (SiO_2, ethyl acetate/n-hexane 1:1): R_f = 0.71.

IR (film): $\tilde{\nu}$ (cm^{-1}) = 1766, 1709, 1657, 1610, 1474, 1406, 1198, 1110, 1019, 914, 845, 809, 767, 696 and 651.

^{1}H-NMR (mixture of stereoisomers, 300 MHz, $CDCl_3$): Main *E*-isomer: δ (ppm): 2.27 (s, 3 H, $COCH_3$), 3.55 (t, J = 7 Hz, 2 H, CH_2Br), 4.22 (t, J = 7 Hz, 2 H, NCH_2), 6.48 (s, 1 H, CH), 7.10 (m, 2 H, CH), 7.26 (m, 2 H, CH), 7.36 (m, 2 H, CH), 7.78 (m, 2 H, CH). **Minor *Z*-isomer:** characteristic peaks only. δ (ppm): 2.22 (s, 3 H, $COCH_3$), 3.05 (t, J = 6 Hz, 2 H, CH_2Br), 3.99 (t, J = 6 Hz, 2 H, NCH_2), 6.71 (s, 1 H, CH).

^{13}C-NMR (mixture of stereoisomers, 75 MHz, $CDCl_3$): Main *E*-isomer: δ (ppm): 20.2 (q, 1 C, CO<u>C</u>H_3), 26.7 (t, 1 C, CH_2Br), 40.0 (t, 1 C, NCH_2), 108.4 (d, 1 C, CH), 114.8 (s, 1 C, Cq), 121.0 (d, 2 C, CH), 122.3 (d, 1 C, CH), 122.4 (d, 1 C, CH), 128.6 (d, 1 C, CH), 129.6 (d, 2 C, CH), 131.0 (s, 1 C, CH), 131.2 (d, 1 C, Cq), 133.8 (s, 1 C, Cq), 135.0 (s, 1 C, Cq), 149.3 (s, 1 C, Cq), 165.5 (s, 1 C, C=O), 168.4 (s, 1 C, C=O). **Minor *Z*-isomer:** quantity too low to assign peaks.

3-Benzylidene-2-(3-bromopropyl)-2,3-dihydro-1*H*-isoindol-1-one (7j)

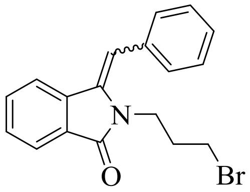

Appearance: pale yellow solid.

TLC (SiO_2, ethyl acetate/n-hexane 1:1): R_f = 0.91.

Melting point: 127°C.

IR (film): $\tilde{\nu}$ (cm^{-1}) = 3148, 1633, 1663, 1574, 1327, 841 and 680.

^{1}H-NMR (mixture of stereoisomers, 300 MHz, $CDCl_3$): Main *E*-isomer: δ (ppm): 2.34 (m, 2 H, CH_2), 3.52 (t, J = 9 Hz, 2 H, CH_2Br), 4.05 (t, J = 9 Hz, 2 H, NCH_2), 6.60 (s, 1 H, CH), 7.29-7.32 (m, 2 H, CH), 7.42-7.46 (m, 6 H, CH), 7.83 (d, J = 9 Hz, 1 H, CH). **Minor *Z*-isomer:** quantity too low to assign peaks.

^{13}C-NMR (mixture of stereoisomers, 75 MHz, $CDCl_3$): Main *E*-isomer: δ (ppm): 30.7 (t, 1 C, CH_2), 31.5 (t, 1 C, CH_2Br), 38.1 (t, 1 C, NCH_2), 110.5 (d, 1 C, CH), 123.2 (d, 1 C, CH), 123.3 (s, 1 C, Cq), 128.0 (d, 1 C, CH), 128.3 (s, 1 C, Cq), 128.4 (d, 2 C, CH), 128.8 (d, 1 C, CH), 129.6 (d, 2 C, Cq), 130.1 (s, 1 C, Cq), 131.7 (d, 1 C, CH), 135.0 (s, 1 C, Cq), 136.1 (s, 1 C, Cq), 166.8 (s, 1 C, C=O). **Minor *Z*-isomer:** quantity too low to assign peaks.

HRMS (mixture of stereoisomers, ESI/MeOH): *m/z*: calcd for $C_{18}H_{16}NOBr$ $(M+Na)^+$: 364.0307, found: 364.0316 ± 3 ppm.

2-(3-Bromopropyl)-3-(4-fluorobenzylidene)-2,3-dihydro-1*H*-isoindol-1-one (7k)

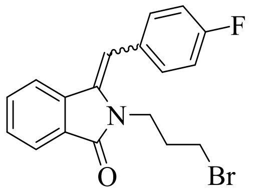

Appearance: yellow oil.

IR (film): $\tilde{\nu}$ (cm^{-1}) = 3129, 1654, 1663, 1582, 1151, 825 and 683.

^{1}H-NMR (mixture of stereoisomers, 300 MHz, $CDCl_3$): Main *E*-isomer: δ (ppm): 2.33 (m, 2 H, CH_2), 3.51 (t, J = 6 Hz, 2 H, CH_2Br), 4.04 (t, J = 6 Hz, 2 H, NCH_2), 6.54 (s, 1 H, CH), 7.14 (dd, J = 6, 3 Hz, 2 H, CH), 7.31-7.47 (m, 5 H, CH), 7.84 (d, J = 6 Hz, 1 H, CH). **Minor *Z*-isomer:** quantity too low to assign peaks.

^{13}C-NMR (mixture of stereoisomers, 75 MHz, $CDCl_3$): Main *E*-isomer: δ (ppm): 27.8 (t, 1 C, CH_2), 30.6 (t, 1 C, CH_2Br), 39.3 (t, 1 C, NCH_2), 110.9 (d, 1 C, CH), 114.6 (d, 1 C, CH), 119.4 (d, 1 C, CH), 123.1 (d, 1 C, CH), 126.9 (d, 1 C, Cq), 129.1 (d, 1 C, CH), 129.4 (d, 1 C, CH), 129.8 (d, 1 C, CH), 130.9 (d, 1 C, CH), 131.7 (d, 1 C, Cq), 132.3 (d, 1 C, CH), 135.1 (s, 1 C, Cq), 138.4 (s, 1 C, Cq), 159.5 (s, 1 C, Cq), 167.8 (s, 1 C, C=O). **Minor *Z*-isomer:** quantity too low to assign peaks.

HRMS (mixture of stereoisomers, ESI/MeOH): *m/z*: calcd for $C_{18}H_{15}NOBrF$ $(M+Na)^+$: 382.0213, found: 382.0215 ± 1 ppm.

2-(3-Bromopropyl)-3-(4-chlorobenzylidene)-2,3-dihydro-1*H*-isoindol-1-one (7l)

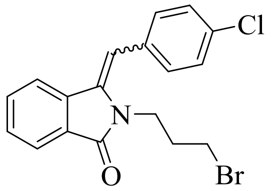

Appearance: yellow oil.

IR (film): $\tilde{\nu}$ (cm^{-1}) = 3156, 1680, 1639, 1580, 1346, 828 and 678.

^{1}H-NMR (mixture of stereoisomers, 300 MHz, $CDCl_3$): Main *E*-isomer: δ (ppm): 2.33 (m, 2 H, CH_2), 3.51 (t, J = 9 Hz, 2 H, CH_2Br), 4.04 (t, J = 9 Hz, 2 H, NCH_2), 6.39 (s, 1 H, CH), 7.34 (m, 3 H, CH), 7.41 (s, 4 H, CH), 7.84 (d, J = 9 Hz, 1 H, CH). **Minor *Z*-isomer:** quantity too low to assign peaks.

^{13}C-NMR (mixture of stereoisomers, 75 MHz, $CDCl_3$): Main *E*-isomer: δ (ppm): 28.1 (t, 1 C, CH_2), 30.6 (t, 1 C, CH_2Br), 41.0 (t, 1 C, NCH_2), 110.4 (d, 1 C, CH), 114.2 (d, 1 C, CH), 119.4 (d, 1 C, CH), 123.3 (d, 1 C, CH), 123.6 (d, 1 C, Cq), 129.2 (d, 1 C, CH), 129.3 (d, 1 C, CH), 130.6 (d, 1 C, CH), 130.9 (d, 1 C, CH), 131.5 (d, 1 C, Cq), 134.3 (d, 1 C, CH), 135.2 (s, 1 C, Cq), 138.4 (s, 1 C, Cq), 159.4 (s, 1 C, Cq), 166.5 (s, 1 C, C=O). **Minor *Z*-isomer:** quantity too low to assign peaks.

HRMS (mixture of stereoisomers, ESI/MeOH): *m/z*: calcd for $C_{18}H_{15}NOBrCl$ $(M+Na)^+$: 397.9918, found: 397.9924 ± 2 ppm.

3-(4-Bromobenzylidene)-2-(3-bromopropyl)-2,3-dihydro-1*H*-isoindol-1-one (7m)

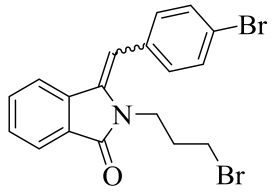

Appearance: yellowish solid.

Melting point: 134-135°C.

IR (film): $\tilde{\nu}$ (cm^{-1}) = 3122, 1676, 1550, 1465, 1338, 832 and 681.

^{1}H-NMR (mixture of stereoisomers, 300 MHz, $CDCl_3$): Main *E*-isomer: δ (ppm): 2.32 (m, 2 H, CH_2), 3.51 (t, J = 6 Hz, 2 H, CH_2Br), 4.03 (t, J = 6 Hz, 2 H, NCH_2), 6.49 (s, 1 H, CH), 7.32 (m, 1 H, CH), 7.35 (m, 2 H, CH), 7.45 (dd, J = 6, 2 Hz, 2 H, CH), 7.57 (d, J = 6 Hz, 2 H, CH), 7.84 (d, J = 6 Hz, 1 H, CH). **Minor *Z*-isomer:** quantity too low to assign peaks.

^{13}C-NMR (mixture of stereoisomers, 75 MHz, $CDCl_3$): Main *E*-isomer: δ (ppm): 30.3 (t, 1 C, CH_2), 32.5 (t, 1 C, CH_2Br), 39.0 (t, 1 C, NCH_2), 109.6 (d, 1 C, CH), 114.9

(d, 1 C, CH), 122.1 (d, 1 C, CH), 125.0 (d, 1 C, Cq), 128.7 (d, 1 C, CH), 129.2 (d, 2 C, CH), 130.9 (d, 2 C, CH), 131.5 (d, 1 C, Cq), 132.6 (d, 1 C, CH), 135.5 (s, 1 C, Cq), 137.1 (s, 1 C, Cq), 145.1 (s, 1 C, Cq), 167.5 (s, 1 C, C=O). **Minor *Z*-isomer:** quantity too low to assign peaks.

HRMS (mixture of stereoisomers, ESI/MeOH): *m/z*: calcd for $C_{18}H_{15}NOBr_2$ $(M+Na)^+$: 441.9413, found: 441.9433 ± 5 ppm.

2-(3-Bromopropyl)-3-(4-methoxybenzylidene)-2,3-dihydro-1*H*-isoindol-1-one (7n)

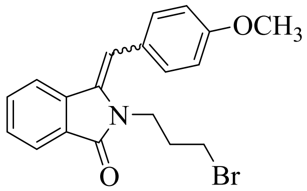

Appearance: yellowish solid.

Melting point: 126°C.

IR (film): $\tilde{\nu}$ (cm^{-1}) = 3145, 1684, 1600, 1567, 1378, 1248, 880 and 649.

^{1}H-NMR (mixture of stereoisomers, 300 MHz, $CDCl_3$): Main *E*-isomer: δ (ppm): 2.30-2.35 (m, 2 H, CH_2), 3.64 (t, J = 6 Hz, 2 H, CH_2Br), 3.90 (s, 3 H, OCH_3), 4.07 (t, J = 6 Hz, 2 H, NCH_2), 6.77 (s, 1 H, CH), 7.06 (d, J = 9 Hz, 2 H, CH), 7.44-7.46 (m, 5 H, CH), 7.79 (d, J = 9 Hz, 1 H, CH). **Minor *Z*-isomer:** quantity too low to assign peaks.

^{13}C-NMR (mixture of stereoisomers, 75 MHz, $CDCl_3$): Main *E*-isomer: δ (ppm): 30.7 (t, 1 C, CH_2), 31.6 (t, 1 C, CH_2Br), 38.0 (t, 1 C, NCH_2), 55.3 (q, 1 C, OCH_3), 110.4 (d, 1 C, CH), 114.2 (d, 1 C, CH), 123.2 (d, 1 C, CH), 123.5 (d, 1 C, Cq), 127.2 (d, 1 C, CH), 127.3 (d, 2 C, CH), 129.2 (d, 2 C, CH), 129.4 (d, 1 C, Cq), 130.2 (d, 1 C, CH), 132.0 (s, 1 C, Cq), 134.1 (s, 1 C, Cq), 159.6 (s, 1 C, Cq), 166.7 (s, 1 C, C=O). **Minor *Z*-isomer:** quantity too low to assign peaks.

HRMS (mixture of stereoisomers, ESI/MeOH): *m/z*: calcd for $C_{19}H_{18}O_2NBr$ $(M+Na)^+$: 394.0413, found: 394.0421 ± 2 ppm.

2-(3-Bromopropyl)-3-(4-methylbenzylidene)-2,3-dihydro-1*H*-isoindol-1-one (7o)

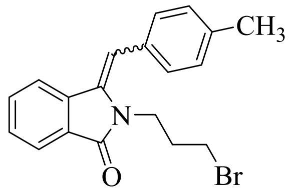

Appearance: yellow oil.

IR (film): $\tilde{\nu}$ (cm^{-1}) = 3101, 1679, 1602, 1581, 1372, 846 and 669.

^{1}H-NMR (mixture of stereoisomers, 300 MHz, $CDCl_3$): Main *E*-isomer: δ (ppm): 2.31-2.36 (m, 2 H, CH_2), 2.43 (s, 3 H, CH_3), 3.51 (t, J = 6 Hz, 2 H, CH_2Br), 4.04 (t, J = 6 Hz, 2 H, NCH_2), 6.58 (s, 1 H, CH), 7.23-7.29 (m, 1 H, CH), 7.32-7.45 (m, 6 H, CH), 7.83 (d, J = 6 Hz, 1 H, CH). **Minor *Z*-isomer:** quantity too low to assign peaks.

^{13}C-NMR (mixture of stereoisomers, 75 MHz, $CDCl_3$): Main *E*-isomer: δ (ppm): 21.3 (q, 1 C, CH_3), 28.3 (t, 1 C, CH_2), 30.6 (t, 1 C, CH_2Br), 41.3 (t, 1 C, NCH_2), 107.1 (d, 1 C, CH), 114.2 (d, 1 C, CH), 119.1 (d, 1 C, CH), 123.0 (d, 1 C, CH), 123.5 (d, 1 C, Cq), 127.9 (d, 1 C, CH), 129.2 (d, 1 C, CH), 130.9 (d, 1 C, CH), 131.3 (d, 1 C, CH), 131.6 (d, 1 C, Cq), 134.5 (d, 1 C, CH), 135.7 (s, 1 C, Cq), 138.0 (s, 1 C, Cq), 141.4 (s, 1 C, Cq), 166.5 (s, 1 C, C=O). **Minor *Z*-isomer:** quantity too low to assign peaks.

HRMS (mixture of stereoisomers, ESI/MeOH): *m/z*: calcd for $C_{19}H_{18}NOBr$ $(M+Na)^+$: 378.0464, found: 378.0471 ± 2 ppm.

2-(3-Bromopropyl)-3-(3-methylbenzylidene)-2,3-dihydro-1*H*-isoindol-1-one (7p)

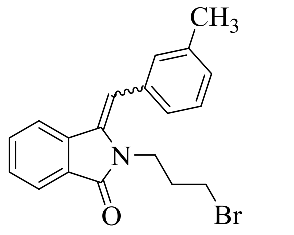

Appearance: yellow oil.

IR (film): $\tilde{\nu}$ (cm^{-1}) = 3098, 1682, 1640, 1580, 1250, 832 and 694.

^{1}H-NMR (mixture of stereoisomers, 300 MHz, $CDCl_3$): Main *E*-isomer: δ (ppm): 2.26 (s, 3 H, CH_3), 2.30 (m, 2 H, CH_2), 3.47 (t, J = 6 Hz, 2 H, CH_2Br), 4.02 (t, J = 9 Hz, 2 H, NCH_2), 6.51 (s, 1 H, CH), 6.92 (d, J = 9 Hz, 1 H, CH), 7.18-7.22 (m, 2 H, CH), 7.24-7.27 (m, 2 H, CH), 7.30-7.38 (m, 2 H, CH), 7.78 (d, J = 9 Hz, 1 H, CH). **Minor *Z*-isomer:** quantity too low to assign peaks.

^{13}C-NMR (mixture of stereoisomers, 75 MHz, $CDCl_3$): Main *E*-isomer: δ (ppm): 20.9 (q, 1 C, CH_3), 28.1 (t, 1 C, CH_2), 30.9 (t, 1 C, CH_2Br), 40.7 (t, 1 C, NCH_2), 109.6 (d, 1 C, CH), 114.5 (d, 1 C, CH), 119.2 (d, 1 C, CH), 123.2 (d, 1 C, CH), 123.4 (d, 1 C, Cq), 129.2 (d, 1 C, CH), 129.6 (d, 1 C, CH), 130.3 (d, 1 C, CH), 130.9 (d, 1 C, CH), 131.1 (d, 1 C, Cq), 134.9 (d, 1 C, CH), 135.5 (s, 1 C, Cq), 138.6 (s, 1 C, Cq), 141.3 (s, 1 C, Cq), 166.5 (s, 1 C, C=O). **Minor *Z*-isomer:** quantity too low to assign peaks.

HRMS (mixture of stereoisomers, ESI/MeOH): *m/z*: calcd for $C_{19}H_{18}NOBr$ $(M+Na)^+$: 378.0464, found: 378.0490 ± 7 ppm.

2-(3-Bromopropyl)-3-(2-methylbenzylidene)-2,3-dihydro-1*H*-isoindol-1-one (7q)

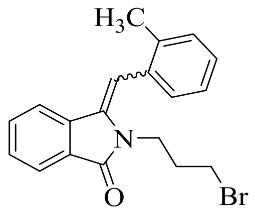

Appearance: yellow oil.

IR (film): $\tilde{\nu}$ (cm^{-1}) = 3137, 1680, 1635, 1578, 1291, 830 and 691.

^{1}H-NMR (mixture of stereoisomers, 300 MHz, $CDCl_3$): Main *E*-isomer: δ (ppm): 2.31-2.36 (m, 2 H, CH_2), 2.40 (s, 3 H, CH_3), 3.51 (t, J = 9 Hz, 2 H, CH_2Br), 4.05 (t, J = 9 Hz, 2 H, NCH_2), 6.58 (s, 1 H, CH), 7.02-7.25 (m, 2 H, CH), 7.27-7.33 (m, 4 H, CH), 7.39-7.45 (m, 1 H, CH), 7.84 (d, J = 9 Hz, 1 H, CH). **Minor *Z*-isomer:** quantity too low to assign peaks.

^{13}C-NMR (mixture of stereoisomers, 75 MHz, CDCl$_3$): Main *E*-isomer: δ (ppm): 21.2 (q, 1 C, CH$_3$), 28.1 (t, 1 C, CH$_2$), 30.6 (t, 1 C, CH$_2$Br), 40.9 (t, 1 C, NCH$_2$), 109.4 (d, 1 C, CH), 114.2 (d, 1 C, CH), 119.4 (d, 1 C, CH), 122.3 (d, 1 C, CH), 123.7 (d, 1 C, Cq), 129.2 (d, 1 C, CH), 129.7 (d, 1 C, CH), 130.1 (d, 1 C, CH), 130.5 (d, 1 C, CH), 131.2 (d, 1 C, Cq), 134.0 (d, 1 C, CH), 135.2 (s, 1 C, Cq), 138.5 (s, 1 C, Cq), 141.7 (s, 1 C, Cq), 166.2 (s, 1 C, C=O). **Minor *Z*-isomer:** quantity too low to assign peaks.

HRMS (mixture of stereoisomers, ESI/MeOH): *m/z*: calcd for $C_{19}H_{18}NOBr$ $(M+Na)^+$: 378.0464, found: 378.0473 ± 2 ppm.

Aminations

3-Benzylidene-2-[2-(diethylamino)ethyl]-2,3-dihydro-1*H*-isoindol-1-one (8a)

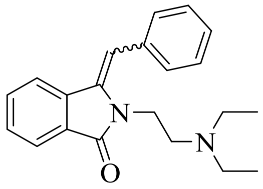

Appearance: beige solid.

Melting point: 43°C.

IR (film): $\tilde{\nu}$ (cm^{-1}) = 3132, 1645, 1612, 1574, 1300, 824 and 691.

^{1}H-NMR (mixture of stereoisomers, 300 MHz, CDCl$_3$): Main *Z*-isomer: δ (ppm): 1.13 (t, J = 9 Hz, 6 H, 2 × CH$_3$), 2.67-2.74 (m, 4 H, N(CH$_2$)$_2$), 2.83 (t, J = 9 Hz, 2 H, NCH$_2$), 4.04 (t, J = 9 Hz, 2 H, NCH$_2$), 6.68 (s, 1 H, CH), 7.33-7.49 (m, 8 H, CH), 7.87 (d, J = 9 Hz, 1 H, CH). **Minor *E*-isomer:** 0.98 (t, J = 7 Hz, 6 H, 2 × CH$_3$), 2.55 (m, 6 H, N(CH$_2$)$_2$), 2.68 (t, J = 8 Hz, 2 H, NCH$_2$), 3.88 (t, J = 8 Hz, 2 H, NCH$_2$), 6.53 (s, 1 H, CH), 7.32-7.34 (m, 8 H, CH), 7.70 (d, J = 9 Hz, 1 H, CH).

^{13}C-NMR (mixture of stereoisomers, 75 MHz, CDCl$_3$): Main *E*-isomer: δ (ppm): 12.1 (q, 2 C, CH$_3$), 39.2 (t, 1 C, CH$_2$N), 47.1 (t, 2 C, N(CH$_2$)$_2$), 50.3 (t, 1 C, NCH$_2$),

106.4 (d, 1 C, CH), 119.3 (d, 1 C, CH), 123.3 (s, 1 C, Cq), 127.7 (d, 1 C, CH), 128.4 (d, 2 C, CH), 128.5 (d, 1 C, CH), 129.0 (d, 2 C, CH), 129.8 (d, 1 C, CH), 131.9 (d, 1 C, CH), 134.9 (s, 1 C, Cq), 135.0 (s, 1 C, Cq), 138.6 (s, 1 C, Cq), 169.0 (s, 1 C, C=O). **Minor *E*-isomer:** 12.3 (q, 2 C, CH_3), 38.2 (t, 1 C, CH_2N), 47.7 (t, 2 C, $N(CH_2)_2$, 50.7 (t, 1 C, NCH_2), 110.6 (d, 1 C, CH), 123.3 (d, 1 C; CH), 123.4 (s, 1 C, Cq), 128.0 (d, 1 C, CH), 128.9 (d, 2 C, CH), 129.4 (d, 1 C, CH), 129.8 (d, 2 C, CH), 130.6 (d, 1 C, CH), 131.6 (d, 1 C, CH), 135.3 (s, 1 C, Cq), 135.4 (s, 1 C, Cq), 136.5 (s, 1 C, Cq), 166.7 (s, 1 C, C=O).

HRMS (mixture of stereoisomers, ESI/MeOH): *m/z*: calcd for $C_{21}H_{24}ON_2$ $(M+Na)^+$: 343.1781, found: 343.1792 ± 3 ppm.

2-[2-(Diethylamino)ethyl]-3-(4-fluorobenzylidene)-2,3-dihydro-1*H*-isoindol-1-one (8b)

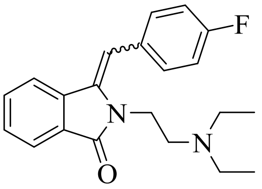

Appearance: beige oil.

IR (film): $\tilde{\nu}$ (cm^{-1}) = 3140, 1680, 1624, 1550, 1154, 819 and 653.

^{1}H-NMR (mixture of stereoisomers, 300 MHz, $CDCl_3$): Main *Z*-isomer: δ (ppm): 1.03 (t, J = 9 Hz, 6 H, 2 × CH_3), 2.60-2.70 (m, 4 H, $N(CH_2)_2$), 2.80 (t, J = 9 Hz, 2 H, NCH_2), 4.00 (t, J = 9 Hz, 2 H, NCH_2), 6.65 (s, 1 H, CH), 7.23-7.27 (m, 2 H, CH), 7.32-7.40 (m, 5 H, CH), 7.84 (d, J = 9 Hz, 1 H, CH). **Minor *E*-isomer:** quantity too low to assign peaks.

^{13}C-NMR (mixture of stereoisomers, 75 MHz, $CDCl_3$): Main *E*-isomer: δ (ppm): 12.1 (q, 2 C, CH_3), 38.9 (t, 1 C, CH_2N), 47.2 (t, 2 C, $N(CH_2)_2$), 50.5 (t, 1 C, NCH_2), 107.1 (d, 1 C, CH), 119.7 (d, 1 C, CH), 123.2 (s, 1 C, Cq), 127.3 (d, 1 C, CH), 128.5 (d, 2 C, CH), 129.0 (d, 1 C, CH), 130.7 (d, 2 C, CH), 131.5 (s, 1 C, Cq), 134.3 (d, 1 C,

CH), 135.3 (s, 1 C, Cq), 138.4 (s, 1 C, Cq), 159.7 (s, 1 C, Cq), 166.5 (s, 1 C, C=O). **Minor *E*-isomer:** quantity too low to assign peaks.

HRMS (mixture of stereoisomers, ESI/MeOH): *m/z*: calcd for $C_{21}H_{23}N_2OF$ $(M+Na)^+$: 361.1687, found: 361.1678 ± 1 ppm.

2-[2-(Diethylamino)ethyl]-3-(4-chlorobenzylidene)-2,3-dihydro-1*H*-isoindol-1-one (8c)

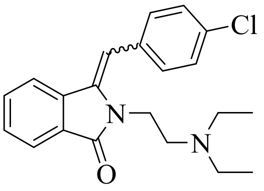

Appearance: beige oil.

IR (film): $\tilde{\nu}$ (cm^{-1}) = 3102, 1632, 1600, 1548, 1378, 855 and 671.

^{1}H-NMR (mixture of stereoisomers, 300 MHz, $CDCl_3$): Main *Z*-isomer: δ (ppm): 1.07 (t, J = 6 Hz, 6 H, 2 × CH_3), 2.61-2.68 (m, 4 H, $N(CH_2)_2$), 2.76 (t, J = 6 Hz, 2 H, NCH_2), 3.97 (t, J = 6 Hz, 2 H, NCH_2), 6.53 (s, 1 H, CH), 7.30-7.45 (m, 7 H, CH), 7.85 (d, J = 9 Hz, 1 H, CH). **Minor *E*-isomer:** quantity too low to assign peaks.

^{13}C-NMR (mixture of stereoisomers, 75 MHz, $CDCl_3$): Main *E*-isomer: δ (ppm): 12.3 (q, 2 C, CH_3), 38.5 (t, 1 C, CH_2N), 47.5 (t, 2 C, $N(CH_2)_2$), 50.4 (t, 1 C, NCH_2), 106.9 (d, 1 C, CH), 119.3 (d, 1 C, CH), 123.9 (s, 1 C, Cq), 127.7 (d, 1 C, CH), 128.0 (d, 2 C, CH), 129.5 (d, 1 C, CH), 130.3 (d, 2 C, CH), 131.8 (s, 1 C, Cq), 134.8 (d, 1 C, CH), 135.7 (s, 1 C, Cq), 138.1 (s, 1 C, Cq), 150.5 (s, 1 C, Cq), 166.5 (s, 1 C, C=O). **Minor *E*-isomer:** quantity too low to assign peaks.

HRMS (mixture of stereoisomers, ESI/MeOH): *m/z*: calcd for $C_{21}H_{23}N_2OCl$ $(M+Na)^+$: 377.1391, found: 377.1400 ± 2 ppm.

2-[2-(Diethylamino)ethyl]-3-(4-bromobenzylidene)-2,3-dihydro-1*H*-isoindol-1-one (8d)

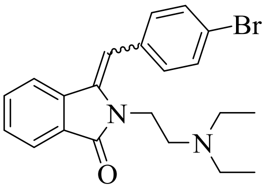

Appearance: beige oil.

IR (film): $\tilde{\nu}$ (cm^{-1}) = 3140, 1663, 1450, 1568, 1370, 860 and 673.

^{1}H-NMR (mixture of stereoisomers, 300 MHz, $CDCl_3$): Main *Z*-isomer: δ (ppm): 1.11 (t, J = 6 Hz, 6 H, 2 × CH_3), 2.65-2.70 (m, 4 H, $N(CH_2)_2$), 2.82 (t, J = 9 Hz, 2 H, NCH_2), 4.14 (t, J = 9 Hz, 2 H, NCH_2), 6.57 (s, 1 H, CH), 7.34-7.41 (m, 5 H, CH), 7.47 (d, J = 9 Hz, 2 H, CH), 7.88 (d, J = 9 Hz, 1 H, CH). **Minor *E*-isomer:** quantity too low to assign peaks.

^{13}C-NMR (mixture of stereoisomers, 75 MHz, $CDCl_3$): Main *E*-isomer: δ (ppm): 12.2 (q, 2 C, CH_3), 38.1 (t, 1 C, CH_2N), 47.3 (t, 2 C, $N(CH_2)_2$), 50.7 (t, 1 C, NCH_2), 106.2 (d, 1 C, CH), 119.0 (d, 1 C, CH), 123.4 (s, 1 C, Cq), 126.9 (d, 1 C, CH), 128.5 (d, 2 C, CH), 129.3 (d, 1 C, CH), 130.7 (d, 2 C, CH), 131.9 (s, 1 C, Cq), 134.3 (d, 1 C, CH), 135.6 (s, 1 C, Cq), 138.9 (s, 1 C, Cq), 145.1 (s, 1 C, Cq), 166.5 (s, 1 C, C=O). **Minor *E*-isomer:** quantity too low to assign peaks.

HRMS (mixture of stereoisomers, ESI/MeOH): *m/z*: calcd for $C_{21}H_{24}BrON_2$ $(M+Na)^+$: 421.0885, found: 421.0882 ± 1 ppm.

2-[2-(Diethylamino)ethyl]-3-(4-methoxybenzylidene)-2,3-dihydro-1*H*-isoindol-1-one (8e)

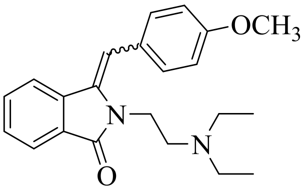

Appearance: beige oil.

IR (film): $\tilde{\nu}$ (cm^{-1}) = 3130, 1679, 1589, 1371, 1268, 830 and 680.

^{1}H-NMR (mixture of stereoisomers, 300 MHz, $CDCl_3$): Main *Z*-isomer: δ (ppm): 1.12 (t, J = 9 Hz, 6 H, 2 × CH_3), 2.65-2.72 (m, 4 H, $N(CH_2)_2$), 2.81 (t, J = 9 Hz, 2 H, NCH_2), 3.92 (s, 3 H, OCH_3), 4.02 (t, J = 9 Hz, 2 H, NCH_2), 6.61 (s, 1 H, CH), 7.01 (d, J = 9 Hz, 2 H, CH), 7.40-7.45 (m, 5 H, CH), 7.87 (d, J = 9 Hz, 1 H, CH). **Minor *E*-isomer:** quantity too low to assign peaks.

^{13}C-NMR (mixture of stereoisomers, 75 MHz, $CDCl_3$): Main *E*-isomer: δ (ppm): 12.0 (q, 2 C, CH_3), 38.0 (t, 1 C, CH_2N), 47.2 (t, 2 C, $N(CH_2)_2$), 50.3 (t, 1 C, NCH_2), 55.4 (q, 1 C, OCH_3), 106.4 (d, 1 C, CH), 119.1 (d, 1 C, CH), 123.1 (s, 1 C, Cq), 127.0 (d, 1 C, CH), 128.8 (d, 2 C, CH), 129.1 (d, 1 C, CH), 130.4 (d, 2 C, CH), 131.9 (s, 1 C, Cq), 134.4 (d, 1 C, CH), 135.8 (s, 1 C, Cq), 138.7 (s, 1 C, Cq), 159.3 (s, 1 C, Cq), 166.5 (s, 1 C, C=O). **Minor *E*-isomer:** quantity too low to assign peaks.

HRMS (mixture of stereoisomers, ESI/MeOH): *m/z*: calcd for $C_{22}H_{26}O_2N_2$ $(M+H)^+$: 351.2067, found: 351.2061 ± 2 ppm. calcd for $C_{22}H_{26}O_2N_2$ $(M+Na)^+$: 373.1886, found: 373.1886 ± 0 ppm.

2-[2-(Diethylamino)ethyl]-3-(4-methylbenzylidene)-2,3-dihydro-1*H*-isoindol-1-one (8f)

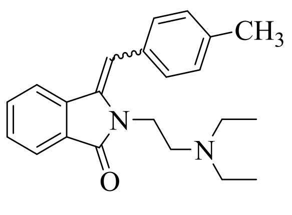

Appearance: beige oil.

IR (film): $\tilde{\nu}$ (cm^{-1}) = 3106, 1634, 1616, 1535, 1257, 820 and 681.

^{1}H-NMR (mixture of stereoisomers, 300 MHz, $CDCl_3$): Main *Z*-isomer: δ (ppm): 1.12 (t, J = 6 Hz, 6 H, 2 × CH_3), 2.31 (s, 3 H, CH_3), 2.65-2.72 (m, 4 H, $N(CH_2)_2$), 2.81

(t, J = 9 Hz, 2 H, NCH_2), 4.05 (t, J = 9 Hz, 2 H, NCH_2), 6.59 (s, 1 H, CH), 7.14 (d, J = 9 Hz, 2 H, CH), 7.35-7.42 (m, 5 H, CH), 7.81 (d, J = 9 Hz, 1 H, CH). **Minor *E*-isomer:** quantity too low to assign peaks.

^{13}C-NMR (mixture of stereoisomers, 75 MHz, $CDCl_3$): Main *E*-isomer: δ (ppm): 12.5 (q, 2 C, CH_3), 20.1 (q, 1 C, CH_3), 38.7 (t, 1 C, CH_2N), 45.7 (t, 2 C, $N(CH_2)_2$), 50.2 (t, 1 C, NCH_2), 107.1 (d, 1 C, CH), 120.6 (d, 1 C, CH), 123.4 (s, 1 C, Cq), 127.7 (d, 1 C, CH), 128.0 (d, 2 C, CH), 129.9 (d, 1 C, CH), 130.7 (d, 2 C, CH), 131.0 (s, 1 C, Cq), 134.7 (d, 1 C, CH), 135.5 (s, 1 C, Cq), 138.5 (s, 1 C, Cq), 143.7 (s, 1 C, Cq), 166.5 (s, 1 C, C=O). **Minor *E*-isomer:** quantity too low to assign peaks.

HRMS (mixture of stereoisomers, ESI/MeOH): *m/z*: calcd for $C_{22}H_{26}N_2O$ $(M+Na)^+$: 357.1937, found: 357.1934 ± 1 ppm.

2-[2-(Diethylamino)ethyl]-3-(3-methylbenzylidene)-2,3-dihydro-1*H*-isoindol-1-one (8g)

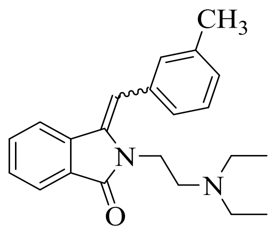

Appearance: beige oil.

IR (film): $\tilde{\nu}$ (cm^{-1}) = 3130, 1626, 1610, 1580, 1287, 829 and 668.

^{1}H-NMR (mixture of stereoisomers, 300 MHz, $CDCl_3$): Main *Z*-isomer: δ (ppm): 1.09 (t, J = 9 Hz, 6 H, 2 × CH_3), 2.40 (s, 3 H, CH_3), 2.63-2.70 (m, 4 H, $N(CH_2)_2$), 2.78 (t, J = 9 Hz, 2 H, NCH_2), 3.99 (t, J = 9 Hz, 2 H, NCH_2), 6.60 (s, 1 H, CH), 7.18-7.25 (m, 2 H, CH), 7.29-7.33 (m, 3 H, CH), 7.38-7.43 (m, 2 H, CH), 7.83 (d, J = 9 Hz, 1 H, CH). **Minor *E*-isomer:** quantity too low to assign peaks.

^{13}C-NMR (mixture of stereoisomers, 75 MHz, $CDCl_3$): Main *E*-isomer: δ (ppm): 12.0 (q, 2 C, CH_3), 20.0 (q, 1 C, CH_3), 38.5 (t, 1 C, CH_2N), 47.2 (t, 2 C, $N(CH_2)_2$),

50.4 (t, 1 C, NCH_2), 106.1 (d, 1 C, CH), 119.1 (d, 1 C, CH), 123.1 (s, 1 C, Cq), 127.0 (d, 1 C, CH), 128.8 (d, 2 C, CH), 129.0 (d, 1 C, CH), 130.9 (d, 1 C, CH), 131.3 (s, 1 C, Cq), 132.7 (d, 1 C, CH), 134.0 (d, 1 C, CH), 135.4 (s, 1 C, Cq), 138.7 (s, 1 C, Cq), 143.1 (s, 1 C, Cq), 166.2 (s, 1 C, C=O). **Minor *E*-isomer:** quantity too low to assign peaks.

HRMS (mixture of stereoisomers, ESI/MeOH): *m/z*: calcd for $C_{22}H_{26}N_2O$ $(M+Na)^+$: 357.1937, found: 357.1945 ± 2 ppm.

2-[2-(Diethylamino)ethyl]-3-(2-methylbenzylidene)-2,3-dihydro-1*H*-isoindol-1-one (8h)

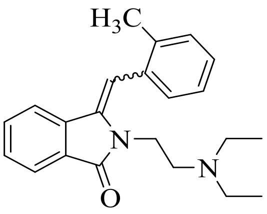

Appearance: beige oil.

IR (film): $\tilde{\nu}$ (cm^{-1}) = 3231, 1650, 1605, 1563, 1233, 816 and 671.

^{1}H-NMR (mixture of stereoisomers, 300 MHz, $CDCl_3$): Main *Z*-isomer: δ (ppm): 1.10 (t, J = 6 Hz, 6 H, 2 × CH_3), 2.31 (s, 3 H, CH_3), 2.65-2.72 (m, 4 H, $N(CH_2)_2$), 2.82 (t, J = 9 Hz, 2 H, NCH_2), 4.00-4.05 (m, 2 H, NCH_2), 6.56 (s, 1 H, CH), 6.98 (m, 2 H, CH), 7.31-7.42 (m, 5 H, CH), 7.82 (d, J = 9 Hz, 1 H, CH). **Minor *E*-isomer:** quantity too low to assign peaks.

^{13}C-NMR (mixture of stereoisomers, 75 MHz, $CDCl_3$): Main *E*-isomer: δ (ppm): 12.0 (q, 2 C, CH_3), 21.3 (q, 1 C, CH_3), 38.9 (t, 1 C, CH_2N), 47.3 (t, 2 C, $N(CH_2)_2$), 50.4 (t, 1 C, NCH_2), 106.7 (d, 1 C, CH), 120.3 (d, 1 C, CH), 123.7 (s, 1 C, Cq), 127.3 (d, 1 C, CH), 128.8 (d, 2 C, CH), 129.3 (d, 1 C, CH), 130.5 (d, 2 C, CH), 133.8 (s, 1 C, Cq), 134.0 (d, 1 C, CH), 135.7 (s, 1 C, Cq), 138.2 (s, 1 C, Cq), 140.8 (s, 1 C, Cq), 166.6 (s, 1 C, C=O). **Minor *E*-isomer:** quantity too low to assign peaks.

HRMS (mixture of stereoisomers, ESI/MeOH): *m/z*: calcd for $C_{22}H_{26}N_2O$ $(M+Na)^+$: 357.1937, found: 357.1934 ± 1 ppm.

3-(4-Acetoxyphenyl)methylene-2-(2-diethylamino)ethyl-2,3-dihydro-1*H*-isoindol-1-one (8i) [4]

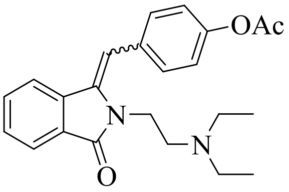

Appearance: beige oil.

^{1}H-NMR (mixture of stereoisomers, 300 MHz, $CDCl_3$): Main *Z*-isomer: δ (ppm): 0.77 (t, J = 6 Hz, 6 H, 2 × CH_3), 2.22 (m, 4 H, $N(CH_2)_2$), 2.31 (s, 3 H, CH_3), 2.54 (t, J = 8 Hz, 2 H, CH_2Br), 3.84 (t, J = 8 Hz, 2 H, NCH_2), 6.66 (s, 1 H, CH), 7.14 (d, J = 9 Hz, 2 H, CH), 7.35 (d, J = 9 Hz, 2 H, CH), 7.50-7.72 (m, 3 H, CH), 7.83 (d, J = 8 Hz, 1 H, CH). **Minor *E*-isomer:** quantity too low to assign peaks.

^{13}C-NMR (mixture of stereoisomers, 75 MHz, $CDCl_3$): Main *E*-isomer: δ (ppm): 12.1 (q, 2 C, CH_3), 21.1 (q, 1 C, CH_3), 39.3 (t, 1 C, CH_2N), 46.9 (t, 2 C, $N(CH_2)_2$), 50.2 (t, 1 C, NCH_2), 105.3 (d, 1 C, CH), 119.0 (d, 1 C, CH), 121.6 (s, 1 C, Cq), 123.2 (d, 1 C, CH), 129.0 (d, 2 C, CH), 130.7 (d, 1 C, CH), 131.7 (d, 2 C, CH), 132.4 (s, 1 C, Cq), 133.8 (d, 1 C, CH), 135.2 (s, 1 C, Cq), 138.4 (s, 1 C, Cq), 150.1 (s, 1 C, Cq), 169.0 (s, 1 C, OC=O), 169.2 (s, 1 C, C=O). **Minor *E*-isomer:** quantity too low to assign peaks.

3-Benzylidene-2-[3-(diethylamino)propyl]-2,3-dihydro-1*H*-isoindol-1-one (8j)

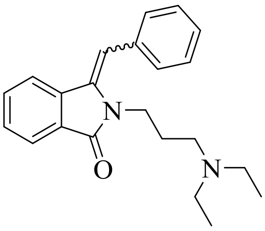

Appearance: beige oil.

IR (film): $\tilde{\nu}$ (cm^{-1}) = 3133, 1642, 1673, 1564, 1324, 849 and 674.

^{1}H-NMR (mixture of stereoisomers, 300 MHz, acetone-d_6): Main *Z*-isomer: δ (ppm): 1.03 (t, J = 9 Hz, 6 H, 2 × CH_3), 1.87-1.96 (m, 2 H, CH_2), 2.52-2.59 (m, 6 H, $N(CH_2)_2$, NCH_2), 3.93 (t, J = 9 Hz, 2 H, NCH_2), 6.58 (s, 1 H, CH), 7.25-7.32 (m, 8 H, CH), 7.83 (d, J = 9 Hz, 1 H, CH). **Minor *E*-isomer:** δ (ppm): 0.96 (t, J = 7 Hz, 6 H, 2 × CH_3), 1.86 (m, 2 H, CH_2), 2.49 (q, J = 7 Hz, 4 H, $N(CH_2)_2$), 2.49 (t, J = 6 Hz, 2 H, NCH_2), 3.87 (t, J = 6 Hz, 2 H, NCH_2), 6.54 (s, 1 H, CH), 7.21 (m, 2 H, CH), 7.32 (m, 5 H, CH), 7.76 (d, J = 8 Hz, 1 H, CH).

^{13}C-NMR (mixture of stereoisomers, 75 MHz, $CDCl_3$): Main *E*-isomer: δ (ppm): 11.6 (q, 2 C, CH_3), 25.9 (t, 1 C, CH_2), 37.8 (t, 1 C, CH_2N), 46.9 (t, 2 C, $N(CH_2)_2$), 50.4 (t, 1 C, NCH_2), 106.8 (d, 1 C, CH), 119.2 (d, 1 C, CH), 123.1 (s, 1 C, Cq), 123.3 (d, 1 C, CH), 126.7 (d, 1 C, CH), 128.2 (d, 2 C, CH), 129.0 (d, 1 C, CH), 130.2 (s, 1 C, Cq), 129.8 (d, 2 C, CH), 131.9 (d, 1 C, CH), 135.2 (d, 1 C, CH), 136.0 (s, 1 C, Cq), 138.5 (s, 1 C, Cq), 168.7 (s, 1 C, C=O). **Minor *E*-isomer:** 10.6 (q, 2 C, CH_3), 25.2 (t, 1 C, CH_2), 36.8 (t, 1 C, NCH_2), 45.8 (t, 2 C, NCH_2), 49.4 (t, 1 C, NCH_2), 109.4 (d, 1 C, CH), 122.0 (d, 1 C, CH), 122.1 (s, 1 C, Cq), 126.7 (d, 1 C, CH), 127.7 (d, 2 C, CH), 128.1 (d, 1 C, CH), 128.6 (d, 2 C, CH), 129.4 (s, 1 C, Cq), 130.3 (d, 1 C, CH), 134.0 (d, 1 C, CH), 134.3 (s, 1 C, Cq), 135.2 (s. 1 C, Cq), 165.6 (s, 1 C, C=O).

HRMS (mixture of stereoisomers, ESI/MeOH): *m/z*: calcd for $C_{22}H_{26}N_2O$ $(M+H)^+$: 335.2118, found: 335.2117 ± 1 ppm. calcd for $C_{22}H_{26}N_2O$ $(M+Na)^+$: 357.1937, found: 357.1943 ± 2 ppm.

2-[3-(Diethylamino)propyl]-3-(4-fluorobenzylidene)-2,3-dihydro-1*H*-isoindol-1-one (8k)

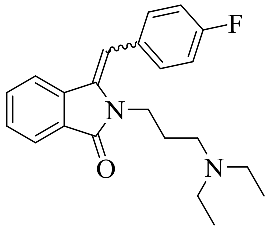

Appearance: beige solid.

Melting point: 40°C.

IR (film): $\tilde{\nu}$ (cm^{-1}) = 3230, 1671, 1648, 1576, 1148, 836 and 671.

^{1}H-NMR (mixture of stereoisomers, 300 MHz, acetone-d_6): Main *Z*-isomer: δ (ppm): 1.02 (t, J = 6 Hz, 6 H, 2 × CH_3), 1.90 (t, J = 6 Hz, 2 H, CH_2), 2.54 (t, J = 6 Hz, 6 H, $N(CH_2)_2$, NCH_2), 3.92 (t, J = 9 Hz, 2 H, NCH_2), 6.54 (s, 1 H, CH), 7.12 (t, J = 9 Hz, 2 H, CH), 7.21 (d, J = 9 Hz, 1 H, CH), 7.30-7.44 (m, 4 H, CH), 7.83 (d, J = 9 Hz, 1 H, CH). **Minor *E*-isomer:** quantity too low to assign peaks.

^{13}C-NMR (mixture of stereoisomers, 75 MHz, $CDCl_3$): Main *E*-isomer: δ (ppm): 11.6 (q, 2 C, CH_3), 25.7 (t, 1 C, CH_2), 38.0 (t, 1 C, CH_2N), 46.3 (t, 2 C, $N(CH_2)_2$), 50.5 (t, 1 C, NCH_2), 106.9 (d, 1 C, CH), 110.7 (d, 1 C, CH), 114.5 (s, 1 C, Cq), 119.7 (d, 1 C, CH), 127.5 (d, 1 C, CH), 128.3 (d, 2 C, CH), 130.4 (d, 1 C, CH), 131.2 (s, 1 C, Cq), 131.7 (s, 1 C, Cq), 134.0 (d, 1 C, CH), 135.8 (d, 1 C, CH), 138.9 (s, 1 C, Cq), 158.5 (s, 1 C, Cq), 167.1 (s, 1 C, C=O). **Minor *E*-isomer:** quantity too low to assign peaks.

HRMS (mixture of stereoisomers, ESI/MeOH): *m/z*: calcd for $C_{22}H_{25}N_2OF$ $(M+H)^+$: 353.2024, found: 353.2031 ± 2 ppm.

2-[3-(Diethylamino)propyl]-3-(4-chlorobenzylidene)-2,3-dihydro-1*H*-isoindol-1-one (8l)

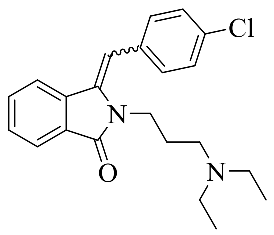

Appearance: beige oil.

IR (film): $\tilde{\nu}$ (cm^{-1}) = 3141, 1648, 1611, 1576, 1332, 841 and 678.

^{1}H-NMR (mixture of stereoisomers, 300 MHz, acetone-d_6): Main *Z*-isomer: δ (ppm): 0.88 (t, J = 9 Hz, 6 H, 2 × CH_3), 1.77 (t, J = 9 Hz, 2 H, CH_2), 2.38-2.45 (m, 6 H, $N(CH_2)_2$, NCH_2), 3.78 (t, J = 9 Hz, 2 H, NCH_2), 6.39 (s, 1 H, CH), 7.12-7.19 (m, 3 H, CH), 7.22-7.25 (m, 2 H, CH), 7.27-7.32 (m, 2 H, CH), 7.69 (d, J = 9 Hz, 1 H, CH). **Minor *E*-isomer:** quantity too low to assign peaks.

^{13}C-NMR (mixture of stereoisomers, 75 MHz, $CDCl_3$): Main *E*-isomer: δ (ppm): 11.8 (q, 2 C, CH_3), 25.3 (t, 1 C, CH_2), 38.1 (t, 1 C, CH_2N), 45.8 (t, 2 C, $N(CH_2)_2$), 50.1 (t, 1 C, NCH_2), 106.1 (d, 1 C, CH), 110.7 (d, 1 C, CH), 114.4 (s, 1 C, Cq), 119.1 (d, 1 C, CH), 127.2 (d, 1 C, CH), 128.7 (d, 2 C, CH), 130.4 (d, 1 C, CH), 130.7 (s, 1 C, Cq), 131.3 (s, 1 C, Cq), 134.9 (d, 2 C, CH), 137.2 (s, 1 C, Cq), 155.7 (s, 1 C, Cq), 166.6 (s, 1 C, C=O). **Minor *E*-isomer:** quantity too low to assign peaks.

HRMS (mixture of stereoisomers, ESI/MeOH): *m/z*: calcd for $C_{22}H_{25}N_2OCl$ $(M+H)^+$: 369.1728, found: 369.1735 ± 2 ppm. calcd for $C_{22}H_{25}N_2OCl$ $(M+Na)^+$: 391.1548, found: 391.1569 ± 5 ppm.

2-[3-(Diethylamino)propyl]-3-(4-bromobenzylidene)-2,3-dihydro-1*H*-isoindol-1-one (8m)

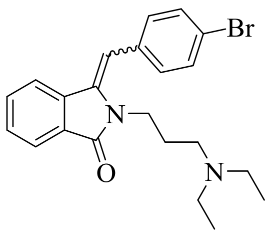

Appearance: redish beige oil.

IR (film): $\tilde{\nu}$ (cm^{-1}) = 3136, 1681, 1538, 1460, 1347, 829 and 688.

^{1}H-NMR (mixture of stereoisomers, 300 MHz, acetone-d$_6$): Main *Z*-isomer: δ (ppm): 1.02 (t, J = 9 Hz, 6 H, 2 × CH_3), 1.90 (t, J = 6 Hz, 2 H, CH_2), 2.52-2.58 (m, 6 H, $N(CH_2)_2$, NCH_2), 3.91 (t, J = 6 Hz, 2 H, NCH_2), 6.49 (s, 1 H, CH), 7.29-7.35 (m, 4 H, CH), 7.43 (t, J = 9 Hz, 1 H, CH), 7.56 (d, J = 9 Hz, 2 H, CH), 7.83 (d, J = 9 Hz, 1 H, CH). **Minor *E*-isomer:** quantity too low to assign peaks.

^{13}C-NMR (mixture of stereoisomers, 75 MHz, $CDCl_3$): Main *E*-isomer: δ (ppm): 11.9 (q, 2 C, CH_3), 25.2 (t, 1 C, CH_2), 38.0 (t, 1 C, CH_2N), 45.3 (t, 2 C, $N(CH_2)_2$), 51.5 (t, 1 C, NCH_2), 107.6 (d, 1 C, CH), 110.4 (d, 1 C, CH), 114.9 (s, 1 C, Cq), 120.3 (d, 1 C, CH), 127.3 (d, 1 C, CH), 128.7 (d, 2 C, CH), 130.1 (d, 1 C, CH), 130.5 (s, 1 C, Cq), 131.8 (s, 1 C, Cq), 134.3 (d, 2 C, CH), 138.9 (s, 1 C, Cq), 145.3 (s, 1 C, Cq), 166.7 (s, 1 C, C=O). **Minor *E*-isomer:** quantity too low to assign peaks.

HRMS (mixture of stereoisomers, ESI/MeOH): *m/z*: calcd for $C_{22}H_{25}N_2OBr$ $(M+Na)^+$: 435.1042, found: 435.1035 ± 2 ppm.

2-[3-(Diethylamino)propyl]-3-(4-methoxybenzylidene)-2,3-dihydro-1*H*-isoindol-1-one (8n)

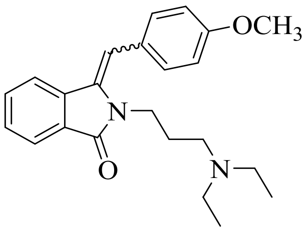

Appearance: beige oil.

IR (film): $\tilde{\nu}$ (cm^{-1}) = 3158, 1662, 1658, 1558, 1362, 1239, 865 and 686.

^{1}H-NMR (mixture of stereoisomers, 300 MHz, $CDCl_3$): Main *Z*-isomer: δ (ppm): 1.06 (t, J = 9 Hz, 6 H, 2 × CH_3), 1.89-1.99 (m, 2 H, CH_2), 2.55-2.62 (m, 6 H, $N(CH_2)_2$, NCH_2), 3.91 (s, 3 H, OCH_3), 3.95 (t, J = 9 Hz, 2 H, NCH_2), 6.60 (s, 1 H, CH), 7.00 (d, J = 9 Hz, 2 H, CH), 7.36-7.41 (m, 5 H, CH), 7.87 (d, J = 9 Hz, 1 H, CH). **Minor *E*-isomer:** quantity too low to assign peaks.

^{13}C-NMR (mixture of stereoisomers, 75 MHz, $CDCl_3$): Main *E*-isomer: δ (ppm): 11.5 (q, 2 C, CH_3), 25.1 (t, 1 C, CH_2), 37.8 (t, 1 C, CH_2N), 46.7 (t, 2 C, $N(CH_2)_2$), 50.4 (t, 1 C, NCH_2), 55.3 (q, 1 C, OCH_3), 106.6 (d, 1 C, CH), 110.5 (d, 1 C, CH), 114.1 (s, 1 C, Cq), 119.2 (d, 1 C, CH), 127.0 (d, 1 C, CH), 128.2 (d, 2 C, CH), 130.3 (d, 1 C, CH), 130.9 (s, 1 C, Cq), 131.4 (s, 1 C, Cq), 134.2 (d, 1 C, CH), 135.6 (d, 1 C, CH), 138.5 (s, 1 C, Cq), 159.1 (s, 1 C, Cq), 167.6 (s, 1 C, C=O). **Minor *E*-isomer:** quantity too low to assign peaks.

HRMS (mixture of stereoisomers, ESI/MeOH): *m/z*: calcd for $C_{23}H_{28}N_2O_2$ $(M+H)^+$: 365.2224, found: 365.2230 ± 2 ppm. calcd for $C_{23}H_{28}N_2O_2$ $(M+Na)^+$: 387.2043, found: 387.2054 ± 3 ppm.

2-[3-(Diethylamino)propyl]-3-(4-methylbenzylidene)-2,3-dihydro-1*H*-isoindol-1-one (8o)

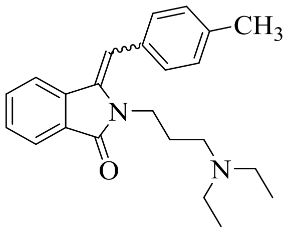

Appearance: beige oil.

IR (film): $\tilde{\nu}$ (cm^{-1}) = 3101, 1682, 1612, 1565, 1361, 850 and 673.

^{1}H-NMR (mixture of stereoisomers, 300 MHz, $CDCl_3$): Main *Z*-isomer: δ (ppm): 1.02 (t, J = 6 Hz, 6 H, 2 × CH_3), 1.91 (m, 2 H, CH_2), 2.42 (s, 3 H, CH_3), 2.52-2.59 (m, 6 H, $N(CH_2)_2$, NCH_2), 3.93 (t, J = 9 Hz, 2 H, NCH_2), 6.58 (s, 1 H, CH), 7.23 (d, J = 9 Hz, 2 H, CH), 7.29-7.36 (m, 4 H, CH), 7.40-7.43 (m, 1 H, CH), 7.82 (d, J = 9 Hz, 1 H, CH). **Minor *E*-isomer:** quantity too low to assign peaks.

^{13}C-NMR (mixture of stereoisomers, 75 MHz, $CDCl_3$): Main *E*-isomer: δ (ppm): 11.9 (q, 2 C, CH_3), 19.5 (q, 1 C, CH_3), 25.7 (t, 1 C, CH_2), 38.8 (t, 1 C, CH_2N), 43.7 (t, 2 C, $N(CH_2)_2$), 51.3 (t, 1 C, NCH_2), 106.1 (d, 1 C, CH), 111.2 (d, 1 C, CH), 114.0 (s, 1 C, Cq), 120.3 (d, 1 C, CH), 127.0 (d, 1 C, CH), 128.4 (d, 2 C, CH), 130.9 (d, 1 C, CH), 131.1 (s, 1 C, Cq), 131.7 (s, 1 C, Cq), 134.4 (d, 1 C, CH), 135.0 (d, 1 C, CH), 138.7 (s, 1 C, Cq), 140.1 (s, 1 C, Cq), 166.7 (s, 1 C, C=O). **Minor *E*-isomer:** quantity too low to assign peaks.

HRMS (mixture of stereoisomers, ESI/MeOH): *m/z*: calcd for $C_{23}H_{28}N_2O$ $(M+Na)^+$: 371.2094, found: 371.2103 ± 3 ppm.

2-[3-(Diethylamino)propyl]-3-(3-methylbenzylidene)-2,3-dihydro-1*H*-isoindol-1-one (8p)

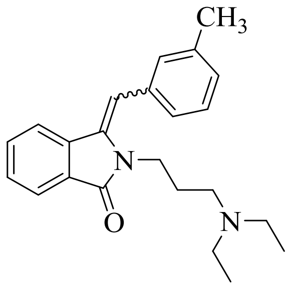

Appearance: beige oil.

IR (film): $\tilde{\nu}$ (cm^{-1}) = 3106, 1685, 1640, 1587, 1243, 834 and 684.

^{1}H-NMR (mixture of stereoisomers, 300 MHz, $CDCl_3$): Main *Z*-isomer: δ (ppm): 1.06 (t, J = 9 Hz, 6 H, 2 × CH_3), 1.97 (m, 2 H, CH_2), 2.34 (s, 3 H, CH_3), 2.55-2.64 (m, 6 H, $N(CH_2)_3$), 3.99 (t, J = 9 Hz, 2 H, NCH_2), 6.51 (s, 1 H, CH), 6.98 (d, J = 6 Hz, 1 H, CH), 7.30-7.46 (m, 6 H, CH), 7.86 (d, J = 6 Hz, 1 H, CH). **Minor *E*-isomer:** quantity too low to assign peaks.

^{13}C-NMR (mixture of stereoisomers, 75 MHz, $CDCl_3$): Main *E*-isomer: δ (ppm): 12.3 (q, 2 C, CH_3), 19.3 (q, 1 C, CH_3), 25.1 (t, 1 C, CH_2), 37.8 (t, 1 C, CH_2N), 46.7 (t, 2 C, $N(CH_2)_2$, NCH_2), 50.4 (t, 1 C, NCH_2), 106.6 (d, 1 C, CH), 110.5 (d, 1 C, CH), 114.1 (s, 1 C, Cq), 119.2 (d, 1 C, CH), 127.0 (d, 1 C, CH), 128.2 (d, 2 C, CH), 130.3 (d, 1 C, CH), 130.9 (s, 1 C, Cq), 131.4 (s, 1 C, Cq), 134.2 (d, 1 C, CH), 135.6 (d, 1 C, CH), 138.1 (s, 1 C, Cq), 140.2 (s, 1 C, Cq), 166.9 (s, 1 C, C=O). **Minor *E*-isomer:** quantity too low to assign peaks.

HRMS (mixture of stereoisomers, ESI/MeOH): *m/z*: calcd for $C_{23}H_{28}N_2O$ $(M+H)^+$: 349.2274, found: 349.2288 ± 4 ppm.

2-[3-(Diethylamino)propyl]-3-(2-methylbenzylidene)-2,3-dihydro-1*H*-isoindol-1-one (8q)

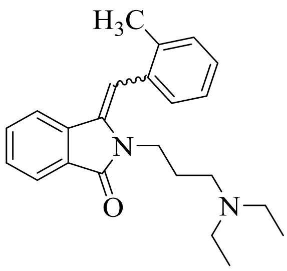

Appearance: beige oil.

IR (film): $\tilde{\nu}$ (cm^{-1}) = 3142, 1681, 1636, 1570, 1284, 854 and 690.

^{1}H-NMR (mixture of stereoisomers, 300 MHz, $CDCl_3$): Main *Z*-isomer: δ (ppm): 1.01 (t, J = 6 Hz, 6 H, 2 × CH_3), 1.93 (m, 2 H, CH_2), 2.41 (s, 3 H, CH_3), 2.48-2.55 (m, 6 H, $N(CH_2)_2$, NCH_2), 4.02 (t, J = 6 Hz, 2 H, NCH_2), 6.57 (s, 1 H, CH), 7.21 (m, 2 H, CH), 7.28-7.36 (m, 4 H, CH), 7.38-7.41 (m, 1 H, CH), 7.86 (d, J = 9 Hz, 1 H, CH). **Minor *E*-isomer:** quantity too low to assign peaks.

^{13}C-NMR (mixture of stereoisomers, 75 MHz, $CDCl_3$): Main *E*-isomer: δ (ppm): 12.0 (q, 2 C, CH_3), 20.1 (q, 1 C, CH_3), 25.2 (t, 1 C, CH_2), 37.1 (t, 1 C, CH_2N), 46.4 (t, 2 C, $N(CH_2)_2$), 50.0 (t, 1 C, NCH_2), 105.6 (d, 1 C, CH), 110.7 (d, 1 C, CH), 114.5 (s, 1 C, Cq), 119.5 (d, 1 C, CH), 127.7 (d, 1 C, CH), 128.0 (d, 2 C, CH), 130.5 (d, 1 C, CH), 130.7 (s, 1 C, Cq), 131.3 (s, 1 C, Cq), 134.2 (d, 1 C, CH), 135.6 (d, 1 C, CH), 138.8 (s, 1 C, Cq), 140.5 (s, 1 C, Cq), 167.1 (s, 1 C, C=O). **Minor *E*-isomer:** quantity too low to assign peaks.

HRMS (mixture of stereoisomers, ESI/MeOH): *m/z*: calcd for $C_{23}H_{28}N_2O$ $(M+H)^+$: 349.2274, found: 349.2288 ± 4 ppm. calcd for $C_{23}H_{28}N_2O$ $(M+Na)^+$: 371.2094, found: 371.2109 ± 4 ppm.

3-Benzylidene-2-[2-(dimethylamino)ethyl]-2,3-dihydro-1*H*-isoindol-1-one (8r)

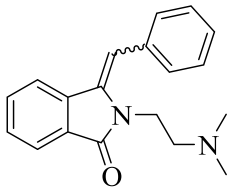

Appearance: yellow oil.

IR (film): $\tilde{\nu}$ (cm^{-1}) = 3154, 1660, 1634, 1576, 1336, 834 and 678.

^{1}H-NMR (mixture of stereoisomers, 300 MHz, $CDCl_3$): Main *Z*-isomer: δ (ppm): 2.42 (s, 6 H, $N(CH_3)_2$), 2.71 (t, J = 6 Hz, 2 H, NCH_2), 4.08 (t, J = 6 Hz, 2 H, NCH_2), 6.58 (s, 1 H, CH), 7.33-7.55 (m, 8 H, CH), 7.88 (d, J = 9 Hz, 1 H, CH). **Minor *E*-isomer:** quantity too low to assign peaks.

^{13}C-NMR (mixture of stereoisomers, 75 MHz, $CDCl_3$): Main *E*-isomer: δ (ppm): 31.1 (t, 1 C, NCH_2), 37.8 (t, 2 C, $N(CH_3)_2$), 56.9 (t, 1 C, CH_2N), 110.3 (d, 1 C, CH), 123.2 (d, 1 C, CH), 123.3 (s, 1 C, Cq), 127.9 (d, 1 C, CH), 128.4 (d, 1 C, CH), 128.8 (d, 2 C, CH), 129.7 (d, 2 C, CH), 132.0 (s, 1 C, Cq), 132.1 (s, 1 C, Cq), 135.2 (d, 1 C, CH), 135.3 (s, 1 C, Cq), 138.2 (d, 1 C, CH), 168.3 (s, 1 C, C=O). **Minor *E*-isomer:** quantity too low to assign peaks.

HRMS (mixture of stereoisomers, ESI/MeOH): *m/z*: calcd for $C_{20}H_{22}ON_2$ $(M+Na)^+$: 329.1624, found: 293.1624 ± 0 ppm.

2-[2-(Dimethylamino)ethyl]-3-(4-fluorobenzylidene)-2,3-dihydro-1*H*-isoindol-1-one (8s)

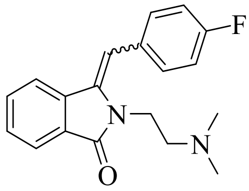

Appearance: beige oil.

IR (film): $\tilde{\nu}$ (cm^{-1}) = 3134, 1687, 1651, 1564, 1154, 828 and 678.

^{1}H-NMR (mixture of stereoisomers, 300 MHz, $CDCl_3$): Main *Z*-isomer: δ (ppm): 2.39 (s, 6 H, $N(CH_3)_2$), 2.57 (t, J = 6 Hz, 2 H, NCH_2), 4.17 (t, J = 6 Hz, 2 H, NCH_2), 6.55 (s, 1 H, CH), 7.29-7.36 (m, 2 H, CH), 7.41-7.50 (m, 5 H, CH), 7.79 (d, J = 9 Hz, 1 H, CH). **Minor *E*-isomer:** quantity too low to assign peaks.

^{13}C-NMR (mixture of stereoisomers, 75 MHz, $CDCl_3$): Main *E*-isomer: δ (ppm): 30.9 (t, 1 C, NCH_2), 37.3 (t, 1 C, NCH_3), 37.7 (t, 1 C, NCH_3), 56.4 (t, 1 C, CH_2N), 109.7 (d, 1 C, CH), 123.0 (d, 1 C, CH), 123.1 (s, 1 C, Cq), 127.9 (d, 1 C, CH), 128.4 (d, 1 C, CH), 128.8 (d, 1 C, CH), 129.7 (d, 2 C, CH), 130.3 (s, 1 C, Cq), 132.1 (s, 1 C, Cq), 135.2 (d, 1 C, CH), 135.2 (s, 1 C, Cq), 135.3 (s, 1 C, Cq), 138.2 (d, 1 C, CH), 168.7 (s, 1 C, C=O). **Minor *E*-isomer:** quantity too low to assign peaks.

HRMS (mixture of stereoisomers, ESI/MeOH): *m/z*: calcd for $C_{19}H_{19}NOF$ $(M+H)^+$: 311.1554, found: 311.1554 ± 1 ppm. calcd for $C_{19}H_{19}NOF$ $(M+Na)^+$: 333.1374, found: 333.1376 ± 1 ppm.

2-[2-(Dimethylamino)ethyl]-3-(4-chlorobenzylidene)-2,3-dihydro-1*H*-isoindol-1-one (8t)

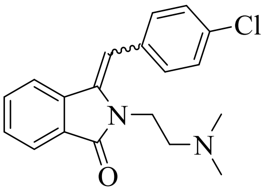

Appearance: yellow oil.

IR (film): $\tilde{\nu}$ (cm^{-1}) = 3102, 1664, 1600, 1548, 1348, 850 and 669.

^{1}H-NMR (mixture or stereoisomers, 300 MHz, $CDCl_3$): Main *Z*-isomer: δ (ppm): 2.40 (s, 6 H, $N(CH_3)_2$), 2.70 (t, J = 6 Hz, 2 H, NCH_2), 4.05 (t, J = 6 Hz, 2 H, NCH_2), 6.57 (s, 1 H, CH), 7.21-7.35 (m, 3 H, CH), 7.21-7.35 (m, 2 H, CH), 7.21-7.35 (m, 2 H, CH), 7.88 (d, J = 9 Hz, 1 H, CH). **Minor *E*-isomer:** quantity too low to assign peaks.

^{13}C-NMR (mixture of stereoisomers, 75 MHz, $CDCl_3$): Main *E*-isomer: δ (ppm): 31.4 (t, 1 C, NCH_2), 37.8 (t, 1 C, NCH_3), 38.0 (t, 1 C, NCH_3), 55.7 (t, 1 C, CH_2N),

110.5 (d, 1 C, CH), 121.1 (d, 1 C, CH), 122.9 (s, 1 C, Cq), 127.4 (d, 1 C, CH), 128.0 (d, 1 C, CH), 128.7 (d, 1 C, CH), 129.0 (d, 2 C, CH), 129.7 (s, 1 C, Cq), 132.4 (s, 1 C, Cq), 135.9 (d, 1 C, CH), 135.9 (s, 1 C, Cq), 136.0 (s, 1 C, Cq), 138.1 (d, 1 C, CH), 168.6 (s, 1 C, C=O). **Minor *E*-isomer:** quantity too low to assign peaks.

HRMS (mixture of stereoisomers, ESI/MeOH): *m/z*: calcd for $C_{19}H_{19}N_2OCl$ $(M+Na)^+$: 349.1078, found: 349.1079 ± 1 ppm.

2-[2-(Dimethylamino)ethyl]-3-(4-methoxybenzylidene)-2,3-dihydro-1*H*-isoindol-1-one (8u)

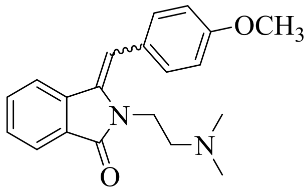

Appearance: beige crystals.

Melting point: 39-41°C.

IR (film): $\tilde{\nu}$ (cm^{-1}) = 3120, 1682, 1580, 1368, 1250, 816 and 660.

^{1}H-NMR (mixture of stereoisomers, 300 MHz, $CDCl_3$): Main *Z*-isomer: δ (ppm): 2.43 (s, 6 H, $N(CH_3)_2$), 2.76 (t, J = 6 Hz, 2 H, NCH_2), 3.62 (s, 3 H, OCH_3), 4.12 (t, J = 6 Hz, 2 H, NCH_2), 6.56 (s, 1 H, CH), 7.47-7.55 (m, 4 H, CH), 7.57-7.60 (m, 2 H, CH), 7.79-7.86 (m, 2 H, CH). **Minor *E*-isomer:** quantity too low to assign peaks.

^{13}C-NMR (mixture of stereoisomers, 75 MHz, $CDCl_3$): Main *E*-isomer: δ (ppm): 31.0 (t, 1 C, NCH_2), 37.7 (t, 1 C, NCH_3), 38.2 (t, 1 C, NCH_3), 56.0 (t, 1 C, CH_2N), 56.5 (q, 1 C, OCH_3), 109.3 (d, 1 C, CH), 122.3 (d, 1 C, CH), 123.4 (s, 1 C, Cq), 127.3 (d, 1 C, CH), 127.9 (d, 1 C, CH), 128.7 (d, 1 C, CH), 129.0 (d, 2 C, CH), 130.7 (s, 1 C, Cq), 132.4 (s, 1 C, Cq), 133.9 (d, 1 C, CH), 134.9 (s, 1 C, Cq), 135.0 (s, 1 C, Cq), 138.7 (d, 1 C, CH), 168.8 (s, 1 C, C=O). **Minor *E*-isomer:** quantity too low to assign peaks.

HRMS (mixture of stereoisomers, ESI/MeOH): *m/z*: calcd for $C_{20}H_{22}N_2O_2$ $(M+H)^+$: 323.1754, found: 323.1761 ± 2 ppm. calcd for $C_{20}H_{22}N_2O_2$ $(M+Na)^+$: 345.1573, found: 345.1575 ± 1 ppm.

2-[2-(Dimethylamino)ethyl]-3-(4-methylbenzylidene)-2,3-dihydro-1*H*-isoindol-1-one (8v)

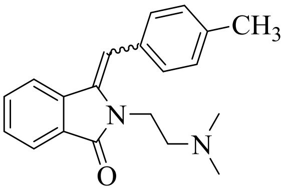

Appearance: beige oil.

IR (film): $\tilde{\nu}$ (cm^{-1}) = 3122, 1651, 1622, 1581, 1288, 847 and 678.

^{1}H-NMR (mixture of stereoisomers, 300 MHz, $CDCl_3$): Main *Z*-isomer: δ (ppm): 2.01 (s, 3 H, CH_3), 2.41 (s, 6 H, $N(CH_3)_2$), 2.68 (t, J = 6 Hz, 2 H, NCH_2), 4.00 (t, J = 6 Hz, 2 H, NCH_2), 6.53 (s, 1 H, CH), 7.35-7.43 (m, 3 H, CH), 7.57-7.60 (m, 3 H, CH), 7.69-7.75 (m, 2 H, CH). **Minor *E*-isomer:** quantity too low to assign peaks.

^{13}C-NMR (mixture of stereoisomers, 75 MHz, $CDCl_3$): Main *E*-isomer: δ (ppm): 20.1 (q, 1 C, CH_3), 31.9 (t, 1 C, NCH_2), 45.4 (t, 2 C, $N(CH_3)_2$), 55.8 (t, 1 C, CH_2N), 110.1 (d, 1 C, CH), 123.0 (d, 1 C, CH), 123.3 (s, 1 C, Cq), 127.5 (d, 1 C, CH), 128.7 (d, 1 C, CH), 129.1 (d, 1 C, CH), 129.2 (d, 1 C, CH), 130.0 (s, 1 C, Cq), 130.1 (s, 1 C, Cq), 130.7 (d, 1 C, CH), 131.4 (d, 1 C, CH), 135.4 (d, 1 C, CH), 135.5 (s, 1 C, Cq), 136.7 (s, 1 C, Cq), 166.5 (s, 1 C, C=O). **Minor *E*-isomer:** quantity too low to assign peaks.

HRMS (mixture of stereoisomers, ESI/MeOH): *m/z*: calcd for $C_{20}H_{22}N_2O$ $(M+H)^+$: 307.1805, found: 307.1806 ± 1 ppm. calcd for $C_{20}H_{22}N_2O$ $(M+Na)^+$: 329.1624, found: 329.1623 ± 1 ppm.

3-Benzylidene-2-[3-(dimethylamino)propyl]-2,3-dihydro-1*H*-isoindol-1-one (8w)

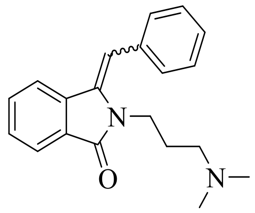

Appearance: beige oil.

IR (film): $\tilde{\nu}$ (cm^{-1}) = 3138, 1640, 1649, 1581, 1329, 852 and 678.

^{1}H-NMR (mixture of stereoisomers, 300 MHz, acetone-d$_6$): Main *Z*-isomer: δ (ppm): 1.91-2.01 (m, 2 H, CH_2), 2.28 (s, 6 H, $N(CH_3)_2$), 2.43 (t, J = 9 Hz, 2 H, NCH_2), 3.98 (t, J = 9 Hz, 2 H, NCH_2), 6.67 (s, 1 H, CH), 7.30-7.48 (m, 8 H, CH), 7.87 (d, J = 9 Hz, 1 H, CH). **Minor *E*-isomer:** quantity too low to assign peaks.

^{13}C-NMR (mixture of stereoisomers, 75 MHz, $CDCl_3$): Main *E*-isomer: δ (ppm): 26.7 (t, 1 C, CH_2), 37.6 (t, 1 C, NCH_2), 45.5 (t, 2 C, $N(CH_3)_2$), 56.9 (t, 1 C, CH_2N), 110.4 (d, 1 C, CH), 123.1 (d, 1 C, CH), 123.2 (s, 1 C, Cq), 127.8 (d, 1 C, CH), 128.7 (d, 1 C, CH), 129.2 (d, 2 C, CH), 129.6 (d, 1 C, CH), 129.9 (s, 1 C, Cq), 130.4 (d, 1 C, CH), 131.4 (d, 1 C, CH), 135.1 (d, 1 C, CH), 135.4 (s, 1 C, Cq), 136.4 (s, 1 C, Cq), 166.7 (s, 1 C, C=O). **Minor *E*-isomer:** quantity too low to assign peaks.

HRMS (mixture of stereoisomers, ESI/MeOH): *m/z*: calcd for $C_{20}H_{22}ON_2$ $(M+Na)^+$: 329.1624, found: 329.1624 ± 1 ppm.

2-[3-(Dimethylamino)propyl]-3-(4-fluorobenzylidene)-2,3-dihydro-1*H*-isoindol-1-one (8x)

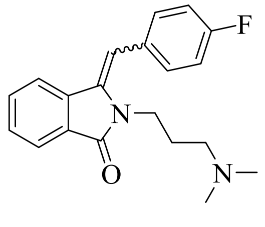

Appearance: beige oil.

IR (film): $\tilde{\nu}$ (cm^{-1}) = 3246, 1633, 1674, 1579, 1145, 843 and 674.

^{1}H-NMR (mixture of stereoisomers, 300 MHz, $CDCl_3$): Main *Z*-isomer: δ (ppm): 1.92-1.97 (m, 2 H, CH_2), 2.30 (s, 6 H, $N(CH_3)_2$), 2.55 (t, J = 9 Hz, 2 H, NCH_2), 3.97 (t, J = 9 Hz, 2 H, NCH_2), 6.57 (s, 1 H, CH), 7.15-7.18 (m, 2 H, CH), 7.22-7.27 (m, 1 H, CH), 7.34-7.45 (m, 4 H, CH), 7.89 (d, J = 9 Hz, 1 H, CH). **Minor *E*-isomer:** quantity too low to assign peaks.

^{13}C-NMR (mixture of stereoisomers, 75 MHz, $CDCl_3$): Main *E*-isomer: δ (ppm): 26.4 (t, 1 C, CH_2), 38.6 (t, 1 C, NCH_2), 43.7 (t, 2 C, $N(CH_3)_2$), 55.4 (t, 1 C, CH_2N), 110.1 (d, 1 C, CH), 120.4 (d, 1 C, CH), 122.7 (s, 1 C, Cq), 127.7 (d, 1 C, CH), 128.3 (d, 1 C, CH), 129.1 (d, 2 C, CH), 129.6 (d, 1 C, CH), 129.8 (s, 1 C, Cq), 130.3 (d, 1 C, CH), 131.4 (d, 1 C, CH), 135.4 (s, 1 C, Cq), 135.9 (s, 1 C, Cq), 157.9 (s, 1 C, Cq), 166.7 (s, 1 C, C=O). **Minor *E*-isomer:** quantity too low to assign peaks.

HRMS (mixture of stereoisomers, ESI/MeOH): *m/z*: calcd for $C_{20}H_{21}N_2OF$ $(M+H)^+$: 325.1711, found: 325.1708 ± 1 ppm. calcd for $C_{20}H_{21}N_2OF$ $(M+Na)^+$: 347.1530, found: 347.1532 ± 1 ppm.

2-[3-(Dimethylamino)propyl]-3-(2-methylbenzylidene)-2,3-dihydro-1*H*-isoindol-1-one (8y)

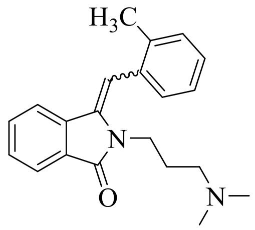

Appearance: beige oil.

IR (film): $\tilde{\nu}$ (cm^{-1}) = 3142, 1687, 1644, 1560, 1280, 864 and 681.

^{1}H-NMR (mixture of stereoisomers, 300 MHz, $CDCl_3$): Main *Z*-isomer: δ (ppm): 1.92-1.97 (m, 2 H, CH_2), 2.12 (s, 3 H, CH_3), 2.24 (s, 6 H, $N(CH_3)_2$), 2.44 (t, J = 6 Hz, 2 H, NCH_2), 3.90 (t, J = 6 Hz, 2 H, NCH_2), 6.50 (s, 1 H, CH), 7.11-7.17 (m, 3 H, CH),

7.35-7.45 (m, 4 H, CH), 7.88 (d, J = 9 Hz, 1 H, CH). **Minor *E*-isomer:** quantity too low to assign peaks.

^{13}C-NMR (mixture of stereoisomers, 75 MHz, $CDCl_3$): Main *E*-isomer: δ (ppm): 20.1 (q, 1 C, CH_3), 26.4 (t, 1 C, CH_2), 36.9 (t, 1 C, NCH_2), 45.4 (t, 2 C, $N(CH_3)_2$), 55.8 (t, 1 C, CH_2N), 110.1 (d, 1 C, CH), 123.0 (d, 1 C, CH), 123.3 (s, 1 C, Cq), 127.5 (d, 1 C, CH), 128.7 (d, 1 C, CH), 129.1 (d, 2 C, CH), 129.2 (d, 1 C, CH), 129.6 (s, 1 C, Cq), 130.7 (d, 1 C, CH), 131.4 (d, 1 C, CH), 135.4 (s, 1 C, Cq), 135.5 (s, 1 C, Cq), 136.7 (s, 1 C, Cq), 166.5 (s, 1 C, C=O). **Minor *E*-isomer:** quantity too low to assign peaks.

HRMS (mixture of stereoisomers, ESI/MeOH): *m/z*: calcd for $C_{21}H_{24}N_2O$ $(M+H)^+$: 321.1961, found: 321.1962 ± 1 ppm. calcd for $C_{21}H_{24}N_2O$ $(M+Na)^+$: 343.1781, found: 343.1784 ± 1 ppm.

Crystallographic data

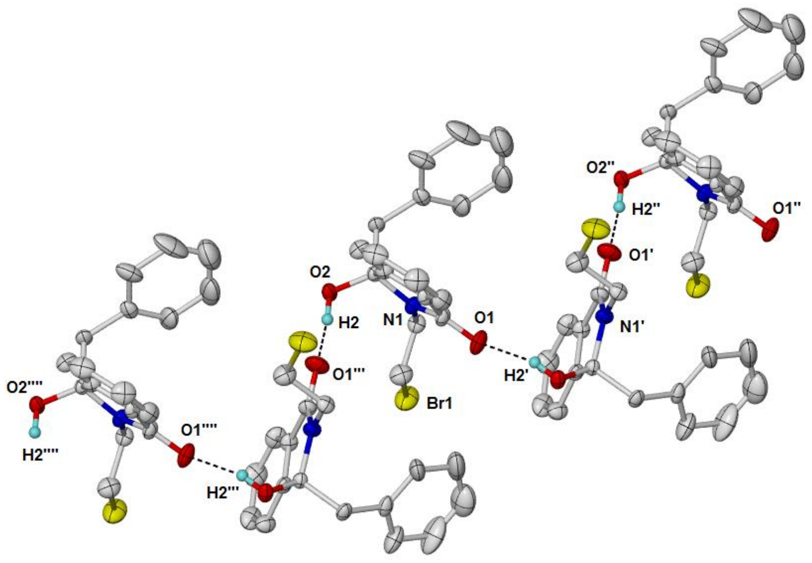

Figure 1: Crystal structure of **3a**.

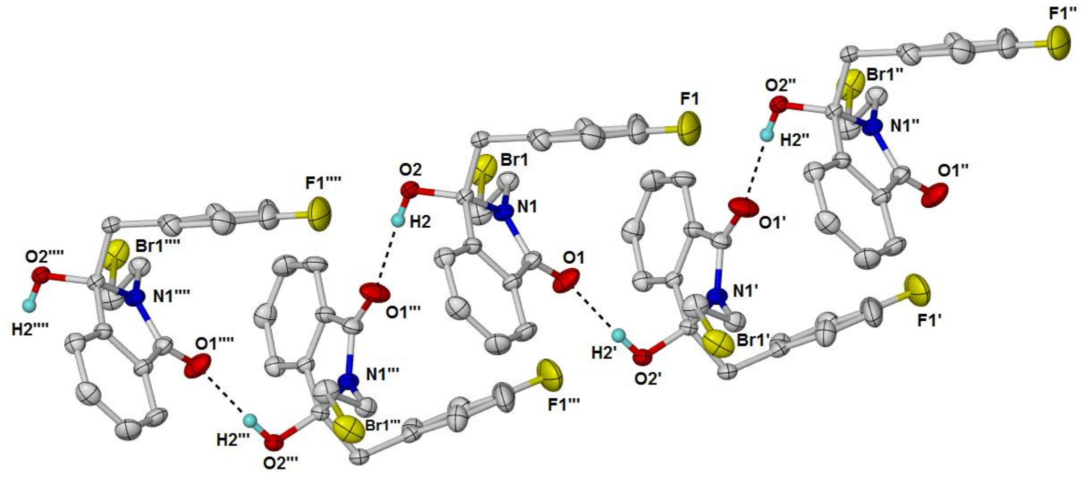

Figure 2: Crystal structure of **3b**.

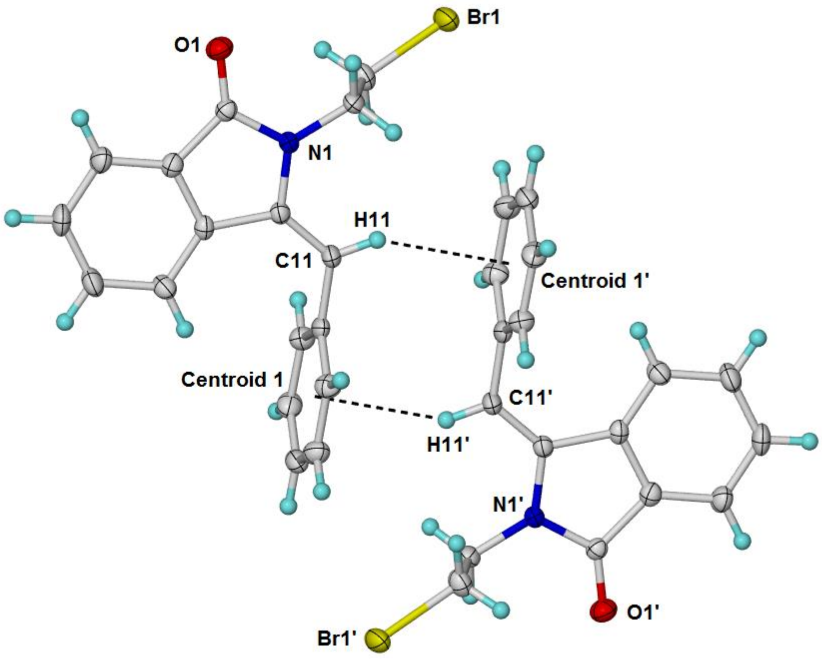

Figure 3: Crystal structure of ***E*-7a**.

Table 1: Key crystallographic data.

**Parameter**	**3a**	**3b**	***E*-7a**	***Z*-8a**
	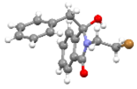	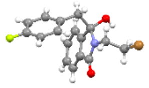	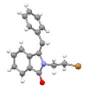	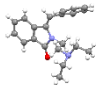
**CCDC**	1417120	1417119	1417121	1417118
**Formula**	$C_{17}H_{16}BrNO_2$	$C_{17}H_{15}BrFNO_2$	$C_{17}H_{14}BrNO$	$C_{21}H_{24}N_2O$
**M_w (g/mol)**	346.21	364.20	328.19	320.42

**a (Å)**	22.295(5)	22.456(5)	8.7086(18)	13.7086(19)
**b (Å)**	9.132(2)	9.0378(18)	8.8445(17)	14.571(2)
**c (Å)**	16.300(4)	16.536(3)	10.632(2)	19.447(3)
**α (°)**	90	90	75.519(4)	90
**β (°)**	104.525(4)	105.133(7)	70.336(4)	107.974(3)
**γ (°)**	90	90	71.145(4)	90
**Volume ($Å^3$)**	3212.6(13)	3239.7(11)	720.5(2)	3694.9(9)
**Z**	8	8	2	8
**δ_{calc} (g/cm^3)**	1.432	1.493	1.513	1.152
**Crystal system**	Monoclinic	monoclinic	triclinic	monoclinic
**Space group**	C 2/c	C 2/c	P -1	C 2/c
**Reflections collected**	3898	3932	3357	4173
**Reflections unique**	1772	2334	2376	2331
**R_1**	0.0757	0.0502	0.0462	0.0571
**wR_2**	0.2540	0.1544	0.1388	0.1654
**Goodness of Fit**	0.975	1.037	1.041	1.032

References

1. Gottlieb, H. E.; Kotlyar, V.; Nudelman, A. *J. Org. Chem.*, **1997**, *62*, 7512-7515.

2. Flack, H. D. *Acta Cryst. A*, **1983**, *39*, 876-881.

3. Bousquet, T.; Fleury, J.-F.; Daïch, A.; Netchitaïlo, P. *Tetrahedron*, **2006**, *62*, 706-715.

4. Couture, A.; Deniau, E.; Grandclaudon, P. *Tetrahedron*, **1997**, *53*, 10313-10330.
